# mTORC1/AMPK responses define a core gene set for developmental cell fate switching

**DOI:** 10.1186/s12915-019-0673-1

**Published:** 2019-07-18

**Authors:** Pundrik Jaiswal, Alan R. Kimmel

**Affiliations:** Laboratory of Cellular and Developmental Biology, National Institute of Diabetes and Digestive and Kidney Diseases, The National Institutes of Health, Bethesda, MD 20892 USA

**Keywords:** RNA-seq, Gene Ontology, Kinase signaling, cAMP, *Dictyostelium*

## Abstract

**Background:**

Kinases mTORC1 and AMPK act as energy sensors, controlling nutrient responses and cellular growth. Changes in nutrient levels affect diverse transcriptional networks, making it challenging to identify downstream paths that regulate cellular growth or a switch to development via nutrient variation. The life cycle of *Dictyostelium* presents an excellent model to study the mTORC1 signaling function for growth and development. *Dictyostelium* grow as single cells in nutrient-rich media, but, upon nutrient withdrawal, growth ceases and cells enter a program for multi-cell development. While nearly half the genome shows gene expression changes upon nutrient removal, we hypothesized that not all of these genes are required for the switch to program development. Through manipulation of mTORC1 activity alone, without nutrient removal, we focused on a core network of genes that are required for switching between growth and development for regulation of cell fate decisions.

**Results:**

To identify developmentally essential genes, we sought ways to promote development in the absence of nutrient loss. We first examined the activities of mTORC1 and AMPK in *Dictyostelium* during phases of rapid growth and starvation-induced development and showed they exhibited reciprocal patterns of regulation under various conditions. Using these as initial readouts, we identified rich media conditions that promoted rapid cell growth but, upon mTORC1 inactivation by rapamycin, led to a growth/development switch. Examination of gene expression during cell fate switching showed that changes in expression of most starvation-regulated genes were not required for developmental induction. Approximately 1000 genes which become downregulated upon rapamycin treatment comprise a cellular growth network involving ribosome biogenesis, protein synthesis, and cell cycle processes. Conversely, the upregulation of ~ 500 genes by rapamycin treatment defines essential signaling pathways for developmental induction, and ~ 135 of their protein products intersect through the well-defined cAMP/PKA network. Many of the rapamycin-induced genes we found are currently unclassified, and mutation analyses of 5 such genes suggest a novel gene class essential for developmental regulation.

**Conclusions:**

We show that manipulating activities of mTORC1/AMPK in the absence of nutrient withdrawal is sufficient for a growth-to-developmental fate switch in *Dictyostelium*, providing a means to identify transcriptional networks and signaling pathways essential for early development.

**Electronic supplementary material:**

The online version of this article (10.1186/s12915-019-0673-1) contains supplementary material, which is available to authorized users.

## Introduction

The mTOR (mechanistic target of rapamycin) kinase is primarily associated with two functionally distinct protein complexes, mTORC1 and mTORC2 [1, 2]. These, in turn, are often suggested to involve separate cellular functions, with mTORC1 being a nutrient sensor and growth regulator and mTORC2 a component of developmental processes [1–4].

Where mTORC1 phosphorylation of S6K and 4EBP1 is essential for protein synthesis and cell growth, the AMP-dependent kinase AMPK serves as a reciprocal nutrient/energy sensor to adjust growth to reducing environmental sustenance [5–7]. Rich media with high concentrations of amino acids and glucose support respectively activation of mTORC1 or inhibition of AMPK. Depletion of amino acids reduces mTORC1 activity [8], and energy-poor conditions activate AMPK, which further inhibits mTORC1 by phospho-activation of the upstream mTORC1 inhibitor TSC2 [9] and phospho-inhibition of raptor [10, 11], an essential mTORC1 subunit. mTORC2 is not a direct target of energy state, although growth factors and metabolic differences influence its activity [1]. mTORC2 primarily impacts cytodifferentiation, with effects on cell polarity and cytoskeletal function [12, 13]. Regardless of apparently separate roles, the two mTOR complexes support overlapping tissue requirements. Active mTORC1 can modulate cell fate choice through balance with mTORC2 [14–18] and promote proliferation of mTORC2-dependent differentiated cells; mTORC2 supports cell survival through glucose homeostasis, membrane function, and migration [19].

The life cycle of *Dictyostelium* presents an excellent model to study the roles of mTORC1 signaling for growth and development [20–23]. In the wild, *Dictyostelium* grows as single cells, where nutrients (e.g., essential amino acids) and enhanced cellular energy status maintain an active state for mTORC1 [24]. However, if environmental nutrients become depleted, mTORC1 activity is suppressed [24] and *Dictyostelium* ceases the growth cell cycle and enters a developmental sequence leading to multi-cell development [21–23]. During *Dictyostelium* development, mTORC2 plays a highly crucial role for inter- and intracellular signaling, cell migration, and aggregation, factors essential for multi-cellular development [22, 24–26].

We hypothesize that manipulation of mTORC1 activity per se, without nutrient removal, might be an essential switch for the growth-to-development transition of *Dictyostelium*, but we needed to avoid global mTOR kinase inhibitors that simultaneously suppressed the developmentally essential mTORC2 [27]. Rapamycin was chosen as an excellent candidate for directed inhibition of mTORC1. The immediate interactive target of rapamycin is cellular protein FKBP12, which in turn binds mTOR to rapidly displace raptor from the complex [28, 29], strongly, but not fully, suppressing phosphorylation of mTORC1 substrates [30, 31]. Rapamycin does not affect mTOR kinase activity per se, and since raptor is not part of mTORC2, rapamycin has no immediate impact on mTORC2. Furthermore, we had previously confirmed the action of rapamycin on mTORC1 in *Dictyostelium* via FKBP12 [24]. Still, chronic (> 10 h) treatment of cells with rapamycin can reduce mTORC2 function, albeit indirectly [24, 32–34].

We first demonstrate fundamentals in *Dictyostelium* for the antagonistic regulations of mTORC1 and AMPK during growth and development and establish essential novel conditions that permit rapid growth of *Dictyostelium*, but also a growth-to-development switch upon direct mTORC1 inhibition by rapamycin in the absence of nutrient withdrawal. Indeed, we show a rapamycin-regulated, dependent downstream path through kinases PKA (protein kinase A, the cAMP-dependent protein kinase) and YakA [35, 36]. Furthermore, we compared gene expression by deep RNA-seq under conditions of starvation and rapamycin-induced development in nutrient-rich media and identify an extensive gene network involved in developmental signal transduction that is upregulated by both starvation and rapamycin and separate downregulated gene classes involved in protein synthesis and in DNA replication and cell division. We suggest that these represent a regulatory core for the growth-to-development transition in *Dictyostelium*. Added to these would be many previously uncharacterized gene classes.

Remarkably, whereas starvation-induced development leads to the rapid change in expression of > 4000 genes, analyses of rapamycin-induced development indicate that half of these gene expression changes are not required for growth/development fate switching. Rather, we suggest that global upregulation of ~ 500 genes and downregulation of ~ 1000 genes define essential early signaling pathways for growth-to-development transition (GDT). Indeed for developmental induction, we show that 5 unclassified genes that were randomly selected from the rapamycin-induced set were essential for early multi-cell development, whereas, in full contrast, none of 10 randomly selected non-rapamycin regulated genes had a significant role for early multi-cell formation or developmental gene expression.

## Results

### Antagonistic actions of mTORC1 and AMPK for nutrient sensing response

*Dictyostelium* grow logarithmically as single cells in nutrient-rich media, but, upon nutrient depletion, growth ceases and cells enter a program for multi-cell development. Since kinases mTORC1 and AMPK function as nutrient and energy sensors in eukaryotes, we wished to determine their relative activities in *Dictyostelium* during rapid growth and starvation-induced development. *Dictyostelium* were grown in nutrient-rich media and washed into a non-nutrient developmental buffer (DB), with mTORC1 and AMPK activities monitored by immunoblot detection of protein-specific phosphorylation/de-phosphorylation kinetics, through 7.5 h of starvation.

**Fig. 1:**
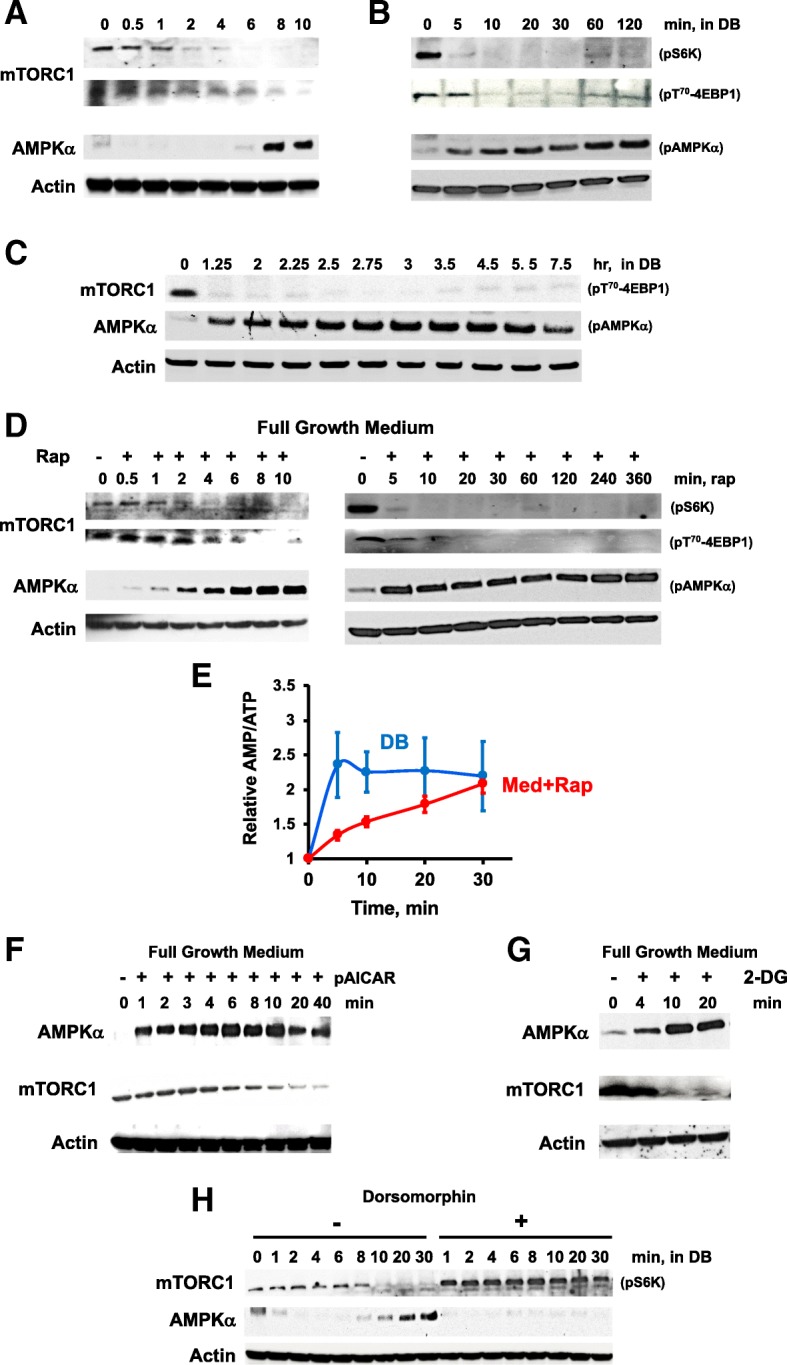
Antagonistic regulation of mTORC1 and AMPK. Nutrient depletion (i.e., in DB) or rapamycin treatment of cells in full growth media inactivates mTORC1 but activates AMPKα. **a**–**c** Nutrient depletion induced de-phosphorylation (i.e., de-activation of mTORC1) of S6K and 4EBP1, but phosphorylation/activation of AMPK. *Dictyostelium* cells were grown to log-phase and transferred to DB in shaking culture. Cell lysates were prepared at times indicated and analyzed by immunoblotting. Similar results were obtained in three independent experiments. **d** Rapamycin treatment of cells in full growth media induces de-phosphorylation of S6K and 4EBP1, but phosphorylation/activation of AMPKα. Cells were grown to log-phase and transferred to a full growth media containing 500 nM of rapamycin. Cell lysates were prepared at times indicated and analyzed by immunoblotting. Similar results were obtained in three independent experiments. **e** Quantification of relative AMP/ATP ratios upon nutrient withdrawal (DB) or rapamycin treatment in full growth media (Med+Rap) in shaking culture. At times indicated, 1 × 10^7^ cells were pelleted and lysed by freeze-thaw. The AMP and ATP levels were measured separately and values represent ratio changes as mean ± standard error. Results are from three independent experiments, with triplicates used for each independent set of experiments. Activation of AMPKα by pAICAR or 2-DG de-activated mTORC1. Log-phase cells in full growth media were treated with 1 mM pAICAR (**f**) or 80 mM 2-DG (**g**) in shaking culture. Cell lysates were prepared at times indicated and analyzed by immunoblotting. Immunoblots are representative of three independent experiments. **h** AMPKα inhibition using dorsomorphin activated mTORC1. AX3 cells were transferred to DB in the presence or absence of 40 μM dorsomorphin in shaking culture. Cell lysates were prepared at times indicated and analyzed by immunoblotting. Immunoblots are representative of three independent experiments

Actively growing cells showed persistent phosphorylation of mTORC1 targets S6K and 4EBP1, but within < 2 min following nutrient removal, we observed de-phosphorylation (i.e., mTORC1 pathway inactivation) of each (Fig. 1a). Full de-phosphorylations were seen within 5–10 min and remained unchanged for 7.5 h of development (Fig. 1b, c). Conversely, AMPK had poor activity (i.e., pAMPKα) in growing cells, but exhibited increased phosphorylation from 5 min after starvation in the non-nutrient buffer, and persistent activation through early development (Fig. 1a–c). Thus, with depletion of nutrient-rich energy supply (e.g., amino acids, glucose) by starvation, AMPK activity rises in parallel to mTORC1 inactivation.

Rapamycin is an immunosuppressant drug that inactivates mTORC1, through cross-binding to cellular protein FKBP12 and TOR kinase, causing dissociation and de-activation of mTORC1. To determine whether mTORC1 action impacted AMPKα, we examined the effects of rapamycin on mTORC1 and AMPK activities in growth phase cells in nutrient-rich media (Fig. 1d). As we had previously shown [24], mTORC1 is rapidly inactivated in *Dictyostelium* by addition of rapamycin to rich growth media, but despite the presence of full nutrient and energy support, rapamycin treatment also promoted the rapid activation of AMPKα (Fig. 1d).

AMPK monitors intracellular energy by sensing changes in relative levels of AMP/ATP. We, thus, investigated whether AMPK activation by rapamycin correlated with an elevated AMP/ATP ratio, quantifying AMP and ATP upon nutrient depletion or rapamycin treatment in nutrient-rich media (Fig. 1e, Additional file 1: Figure S1). Upon starvation (DB), AMP/ATP is rapidly elevated, explaining AMPKα activation by nutrient withdrawal. Rapamycin treatment of cells in nutrient-rich media (Med+Rap) also causes an increase in AMP/ATP, albeit at a slower absolute rate than during starvation. Still, within 20 min, AMP/ATP ratios increase > 2-fold in both starved and rapamycin-treated cells (Fig. 1e, Additional file 1: Figure S1). The reciprocal actions of mTORC1 and AMPK are poised at the junction of *Dictyostelium* growth and development, perhaps serving as essential sensors for the transition between the two cellular states. In addition, there appears to be reciprocal metabolic interaction between mTORC1 and AMPK, since AMPKα is rapidly activated upon direct inhibition of mTORC1 by rapamycin, even in the presence of rich energy nutrient sources.

### AMPK reciprocally regulates the mTORC1 activity

In other systems, activated AMPK can inhibit mTORC1 through phospho-activation of TSC2, an inhibitor of the mTORC1 activator Rheb, and effective phospho-inhibition of the mTORC1 subunit raptor [9–11]. Although *Dictyostelium* has a TSC2/Rheb pathway for mTORC1 regulation, its role is very limited compared to mammalian systems, with only minimal growth differences between WT and *tsc2*- or *rheb*-null cells [24]; effects of this pathway on growth are better observed with decreased or increased sensitivity to rapamycin, for *tsc2*- or *rheb*-null cells, respectively, compared to WT (Additional file 2: Figure S2). Furthermore, *Dictyostelium* TSC2 lacks an AMPKα target site [24]. However, we have observed starvation- and rapamycin-induced phosphorylation of raptor at a predicted AMPKα substrate site for *Dictyostelium* raptor [11], consistent with AMPKα inhibiting mTORC1 by raptor targeting [10, 11].

We sought to modulate AMPKα activity to assess the effects of cross-talk on mTORC1. First, we developed conditions that activated AMPKα in the presence of a rich energy source. pAICAR (phospho 5-aminoimidazole-4-carboxamide ribonucleotide) is a poorly metabolized AMP analog that accordingly increases the activity of AMPK and acts similarly in *Dictyostelium* [37]. 2-Deoxy-d-glucose (2-DG) is a glucose analog that competitively blocks glycolysis, elevates AMP/ATP, and, thus, is similarly an activator of AMPKα. To discern whether active AMPKα can reciprocally suppress mTORC1 activity, we stimulated AMPK activity in growing cells in nutrient-rich media using pAICAR or 2-DG and assayed mTORC1 readout 4EBP1. As seen, AMPKα becomes rapidly phosphorylated during growth by the response to pAICAR or 2-DG, with a corresponding de-phosphorylation of 4EBP1 (Fig. 1f, g). Next, we studied the mTORC1 activity in nutrient-depleted cells that are inhibited for activation of AMPKα. Under standard starvation conditions, mTORC1 activity is reduced as AMPKα phosphorylation increases. However, starved cells treated with dorsomorphin, an inhibitor for AMPKα, were not only inhibited for AMPKα activation during nutrient withdrawal, but mTORC1 inactivation was also suppressed (Fig. 1h).

Although we recognize that kinetics for reciprocal inactivation/activation of mTORC1/AMPK do not describe an immediate mechanistic path and that dorsomorphin can have non-AMPK affects, collectively, the data suggest that the complex cross-talk and interplay of mTORC1/AMPK define conditions to transit *Dictyostelium* from growth phase to development [i.e., growth-to-development transition (GDT)]. To understand the regulatory mechanism under mTORC1/AMPK control, we sought conditions that modulate mTORC1/AMPK to promote development, but in the absence of nutrient depletion.

### mTORC1 inhibition by rapamycin slows cell growth in nutrient-rich media but is insufficient to induce programmed development

Since rapamycin treatment of growing cells in nutrient-rich media mimicked the effects of starvation on the regulation of the mTORC1 and AMPK activities, we pursued whether developmental aspects of the *Dictyostelium* life cycle could be induced by rapamycin-impaired nutrient sensing. Thus, we compared cell growth rates and developmental potential of cells in nutrient-rich media treated with and without rapamycin.

**Fig. 2:**
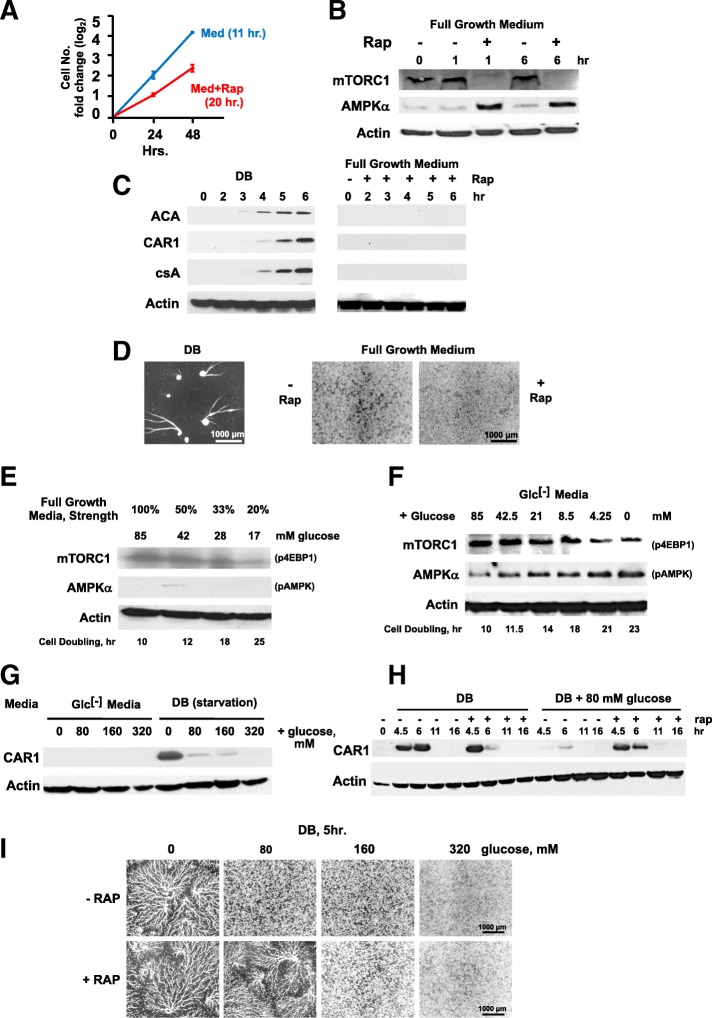
Nutrient effects on kinase activities and developmental induction. **a** Cell growth rates in full growth media without (Med) or with 500 nM rapamycin (Med+Rap). Doubling times represent mean ± standard error from triplicates in an independent experiment (*N* = 3). **b** p4EBP1 and pAMPKα of adhered cells in full growth media in the presence and absence of 500 nM rapamycin. Log-phase cells were adhered to plate wells in fresh full growth media. After 2 h, fresh media were replenished with or without 500 nM rapamycin. Samples were taken at various times and analyzed by immunoblotting. Nutrient withdrawal (DB), but not rapamycin, induced expression of developmental genes and multi-cell formation. Developmental protein expression (**c**) and aggregation (**d**) were examined in adhered cells upon nutrient removal (DB) or in full growth media containing rapamycin. **c** Immunoblots represent three independent experiments. **e** Log-phase cells in full growth media were transferred to indicated strengths of diluted full growth media and growth rates measured. After 24 h, immunoblotting was performed and represent three independent experiments. **f** Log-phase cells in full growth media were transferred to indicated strengths of glucose-free (Glc^[-]^) media, supplemented with varying concentrations of glucose. Growth rates were then measured. After 24 h, samples were analyzed by immunoblotting. Immunoblots are representative of three independent experiments. **g** Adhered log-phase cells in DB or Glc^[-]^ media containing indicated concentrations of glucose. After 5 h, samples analyzed by immunoblotting. Immunoblots represent three independent experiments. **h** Adhered log-phase cells in DB with or without 80 mM glucose and/or rapamycin as indicated. At various times, samples were analyzed by immunoblotting. Immunoblots represent three independent experiments. **i** Adhered log-phase cells in DB with or without mM glucose and/or rapamycin as indicated. After 5 h, cells were visualized for developmental aggregation

Although rapamycin reduces mTORC1 and elevates pAMPKα in cells within nutrient-rich media, growth rates were only reduced by ~ 2-fold (Fig. 2a, see also Additional file 2: Figure S2 [24, 26]. To examine the effects of rapamycin on development, we monitored mTORC1/AMPK activities and expression of early developmental protein markers, ACA (adenylyl cyclase A [38]), CAR1 (the primary receptor for cAMP, 3′,5′-cyclic adenosine monophosphate, during aggregation [21, 39]), and csA (contact site A) in cells adhered to a solid substratum through 6 h. First, we confirmed that rapamycin treatment reciprocally regulated the activities of mTORC1 and AMPKα in cells adhered in the presence of full growth media (Fig. 2b). Yet, although ACA, CAR1, and csA are induced to high levels following full nutrient withdrawal (i.e., in DB), none is expressed in rapamycin-treated cells in the presence of full nutrient-rich media (Fig. 2c). Further, whereas cells adhered in a buffer (DB) will initiate development and form multi-cellular aggregates, cells treated with rapamycin in full growth media do not show developmental signal relay/response and remain as single cells (Fig. 2d). Thus, while rapamycin-impaired nutrient sensing may slow cell growth, it does not fully suspend growth and promote a transition to development. In mammalian cells, rapamycin does not fully suppress phosphorylation of all mTORC1 targets, with dependence to elevated environmental nutrients [30, 31]. We speculate that, as with mammalian cells, the levels of nutrients in rich growth media (e.g., 85 mM glucose) may explain the partial sensitivity of *Dictyostelium* to inhibition by rapamycin.

### Nutrient dissection for inhibition of development

To examine nutrient level effects on *Dictyostelium* development, we first diluted full growth medium to various (20–100%) strengths and compared inherent mTORC1 and AMPK activities and cell growth rates (Fig. 2e) in shaking cultures. We also used a glucose-free (Glc^[-]^) medium that retained full levels of all other nutrient, and additionally titrated in glucose from 0 to 100% for evaluation of effects on kinase activities and growth rates (Fig. 2f). Full growth medium is sufficiently rich that dilution to 0.2× (i.e., 17 mM glucose) only reduces growth rates in shaking culture by two-fold (Fig. 2e) and with minimal effect on mTORC1 and AMPK activities. Likewise, with all other nutrient concentrations unchanged, cells still grow at a 30–50% rate in the absence of glucose, using (Glc^[-]^) medium (Fig. 2f).

Using CAR1 expression as a readout for starvation-induced early development, we show that, although cells adhered in nutrient-free developmental buffer (DB) will express CAR1 to high levels, addition of just a standard glucose (~ 80 mM) concentration to DB or a standard nutrient mix (e.g., amino acids) in the absence of glucose (i.e., using Glc^[-]^ medium) is sufficient to block CAR1 expression and multi-cell development (Fig. 2g).

We also show that glucose or only select essential amino acids are individually able to re-activate mTORC1 or de-activate pAMPKα in starved *Dictyostelium*, where mTORC1 had been de-activated and pAMPKα induced (Additional file 3: Table S1). In addition, since glucose-supplemented DB inhibits mTORC1 inactivation and blocks induction of developmental gene *CAR1* (Fig. 2g), we hypothesized that rapamycin treatment might rescue the development of these glucose-treated cells, lacking amino acids and other nutrients. CAR1 is highly expressed in starved (DB) cells (without glucose and regardless of the presence of rapamycin), but the addition of glucose to DB significantly suppressed CAR1 expression (Fig. 2h). However, rapamycin treatment of DB cells supplemented with glucose was sufficient to induce CAR1 expression (Fig. 2h).

These nutrient level effects are further emphasized when morphological development is visualized. Cells starved in DB undergo cell-cell signaling and chemotaxis, seen as streams of aggregating cells (Fig. 2i). But, the addition of glucose to the developmental media (DB) blocks multi-cell, developmental aggregate formation; cell-cell communication and chemotaxis are inhibited. The inhibitory effect of 80 mM glucose on the initiation of development was fully reversed upon treatment with rapamycin. However, increasing glucose levels beyond that in standard media antagonized the action of rapamycin and prevented development (Fig. 2i). These data emphasize the balance between nutrient levels and rapamycin inhibition of mTORC1 in control of development and suggest that we might define nutrient conditions that support rapid growth, but which remain sensitive for rapamycin-induced development via inhibition of mTORC1 and activation of AMPK.

**Fig. 3:**
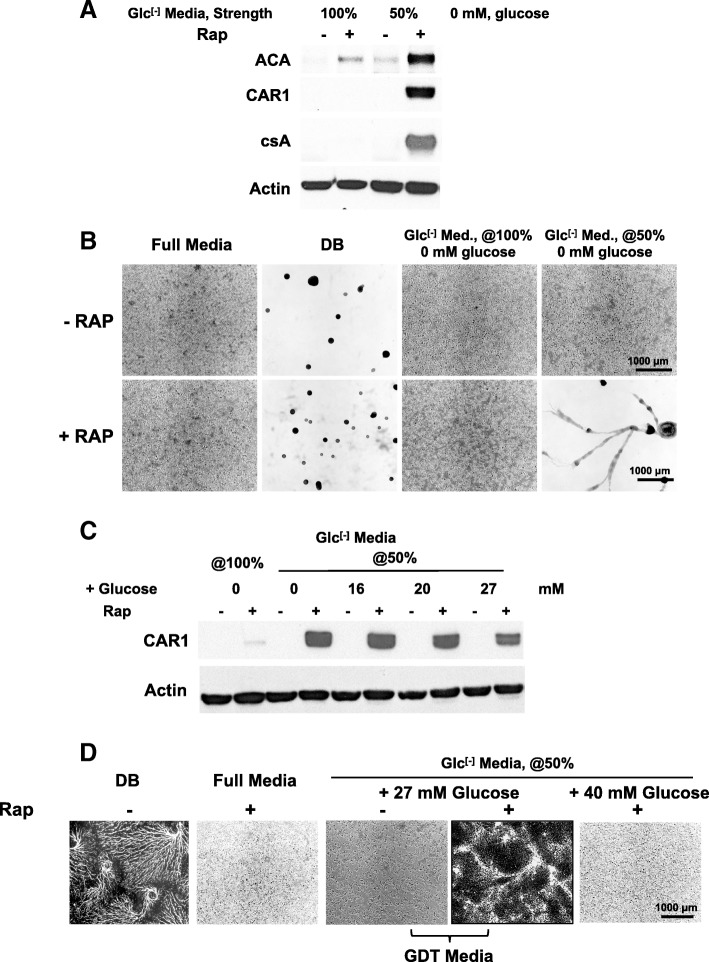
Identification of media for rapid growth but permissive to rapamycin-induced development. **a** Log-phase cells in full growth media were adhered to cell well plates in Glc^[-]^ media at 100% or 50% strength; after 2 h, fresh media were replaced with and without rapamycin. After 10 h, were analyzed by immunoblotting. Immunoblots are representative of three independent experiments. **b** Log-phase cells in full growth media were adhered to cell well plates in full media. After 2 h, media were replaced with fresh full media, DB, Glc^[-]^ media at 100%, or Glc^[-]^ media at 50%, with and without rapamycin. After 10 h, were visualized for developmental aggregation. **c** Log-phase cells in full growth media were adhered to cell well plates in Glc^[-]^media at 100% or 50% strength, with varying concentrations of glucose. After 2 h, media were replaced with and without rapamycin. After 10 h, samples were analyzed by immunoblotting. Immunoblots are representative of three independent experiments. **d** Log-phase cells in full growth media were adhered to cell well plates in full media. After 2 h, media were replaced with fresh full media, DB, Glc^[-]^ media at 50%, with glucose as indicated, and with or without rapamycin as indicated. Developmental aggregation was visualized. GDT media is defined as 50% Glc^[-]^ Media + 27 mM glucose

We next studied the effects of rapamycin on the development of adhered cells in varying concentrations of nutrient media lacking glucose (Glc^[-]^ Media). Although cells were grown in full-strength, glucose-free (Glc^[-]^) media are unable to develop in the absence or presence of rapamycin; dilution of Glc^[-]^ media to 50% permitted rapamycin-induced development (Fig. 3a). We also show that while multi-cell formation is not supported in 50% Glc^[-]^ media (Fig. 3b), the developmental aggregation will occur with rapamycin treatment of 50% Glc^[-]^ media (Fig. 3b).

Since 50% Glc^[-]^ media does not support high growth rates (doubling time > 25 h), we titrated glucose into 50% strength Glc^[-]^ media to identify conditions that are maximized for growth but still permissive to rapamycin-induced development. Addition of glucose at 27 mM to 50% Glc^[-]^ media strongly supported cell growth with doubling times of ~ 13 h, ~ 80% the rate of cells grown in complete media (see Fig. 2a). Strikingly, these rapidly growing cells also entered a normal developmental cycle upon treatment with rapamycin; we observed rapamycin-induced CAR1 expression in 50% Glc^[-]^ media supplemented with glucose to 27 mM (Fig. 3c) and multi-cellular development (Fig. 3d). Increasing glucose by only 13 mM, to 40 mM, was sufficient to antagonize the effects of rapamycin on development (Fig. 3d). These data define specific media conditions [i.e., GDT media (50% Glc^[-]^ media + 27 mM glucose)] that support rapid cell growth, but where inhibition/activation of mTORC1/AMPK is sufficient to shift the cell state from the growth phase to development, countering the inhibitory effects of an external nutrient and energy supply.

### Rapamycin induces a growth-to-development transition in rapidly growing cells involving the YakA/PKA/ACA/CAR1 pathway

*Dictyostelium* development is dependent upon an oscillating signal relay system for cAMP synthesis, secretion, degradation, and response. Previous studies had demonstrated roles of YakA, PKA, and ERK2 protein kinases in establishing cAMP signaling [35, 36, 40–42]. Starvation-activated YakA is suggested to upregulate the expression of PKA subunits, which in turn induces expression of adenylyl cyclase A, the primary enzyme for cAMP production during early development [38]. Activated ERK2 suppresses the activity of the cAMP-degrading RegA phosphodiesterase. Thus, YakA, PKA, ACA, ERK2, and others are essential to promote GDT and drive early *Dictyostelium* development, through modulation of the cAMP signal/response pathway.

**Fig. 4:**
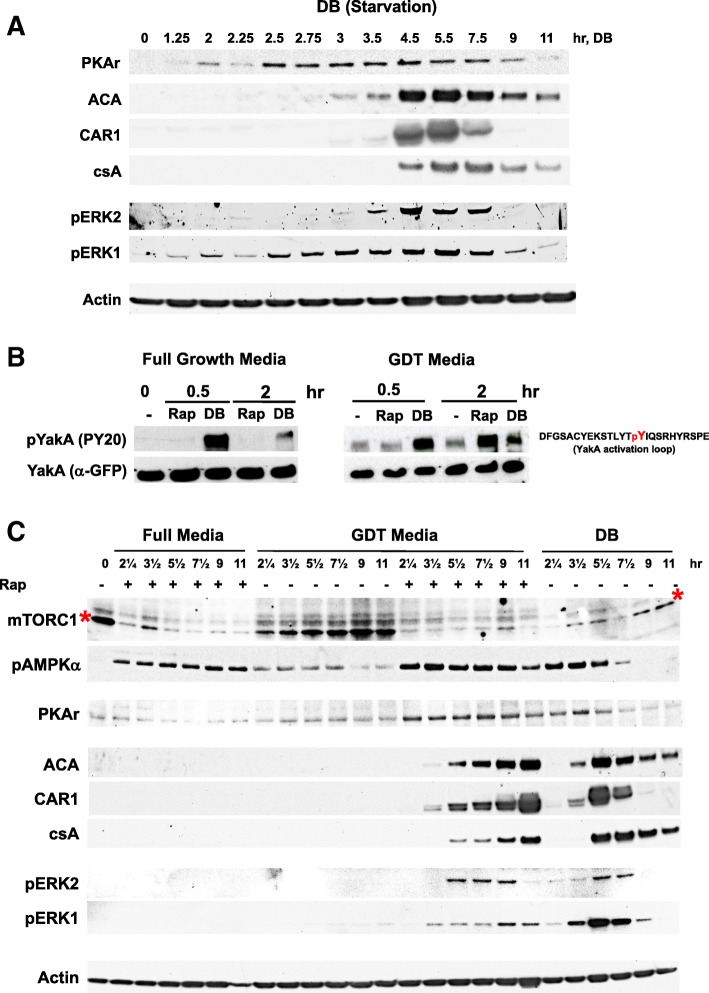
Comparative expression parameters for rapamycin-induced development. **a** Log-phase cells in full growth media were adhered to cell well plates; after 2 h, fresh media were replaced with DB, and samples were analyzed by immunoblotting. Immunoblots are representative of three independent experiments. **b** YakA^−^/YakA-GFP^OE^ cells in full growth media were adhered to a 6-well plate and in full media (left panel) or GDT media (right panel). Left panel—after 2 h, full media were replaced with fresh full media with or without rapamycin or with DB. Right panel—after 2 h, GDT media were replaced with fresh GDT media with or without rapamycin or with DB. Cell lysates were prepared at times indicated. Phosphorylation status of YakA was monitored by immunoblotting with α-GFP and PY20 to GFP-YakA. Immunoblots are representative of three independent experiments. **c** Log-phase cells in full growth media were adhered to cell well plates, in full media or GDT media. After 2h, full media were replaced with fresh media, and GDT media replaced with either fresh GDT media or with DB, all with or without rapamycin as indicated. Samples were taken for immunoblotting for protein gene expression as indicated. Immunoblots shown are representative of three independent experiments

Expression of PKA subunits increases very early following starvation in DB (e.g., Fig. 4a), followed by PKA-dependent induction of development markers ACA, CAR1, and csA (Fig. 4a). Activation kinetics for pERK1/pERK2, ACA, and CAR1 follow PKA expression and regulate synthesis/accumulation of and response to secreted extracellular cAMP during multi-cell formation (Fig. 4a).

A genetic and biochemical pathway linking these has been suggested. PufA is a translational repressor of PKA [35]. YakA suppresses the action of PufA, relieving PKA from inhibition. In turn, PKA is required to activate ACA expression. Accordingly, we show by RNA-seq (see below) that general patterns for upregulated YakA/PKA/ACA/CAR1 expression by starvation are mimicked in GDT media cells treated with rapamycin (Additional file 4: Table S2). None of the genes shows altered expression upon treatment of full media with rapamycin.

YakA appears critical for the GDT expression shift [36]. YakA is a member of the DYRK (dual-specificity tyrosine phosphorylation-regulated kinase) family, where activation is reflected by levels of a tyrosine phosphorylation within the kinase activation loop (see Fig. 4b). In an exploratory phospho-proteome analysis using MS/MS of *Dictyostelium* subjected to starvation (unpublished data), we observed a > 1.5-fold phospho-YakA peptide STLYTpYIQSR abundance ratio increase within 15 min after starvation (Additional file 5: Figure S3). To quantify pYakA activation further, we expressed YakA-GFP in cells lacking YakA and monitored YakA tyrosine phosphorylation by immunoblot assay during growth, in response to starvation and in response to rapamycin in full growth media or GDT media. We consistently observed that cells in the growth phase (i.e., in full growth medium, in full growth medium + rapamycin, or in GDT medium without rapamycin) have very low levels of pTyr YakA-GFP. However, developmental induction through starvation or rapamycin-treated GDT medium elicited strong pTyr YakA-GFP, consistent with pYakA activation response for GDT (Fig. 4b). pYakA peak activation response to DB starvation is rapid, before diminishing, with the rapamycin-induction response delayed 1–2 h in comparison (see below).

We wished to more fully compare early developmental timing events for mTORC1/AMPK activities and developmental marker expression among various culture treatments. We observed inactivation of mTORC1 and activation of AMPKα in DB and rapamycin-treated full media or GDT media, but not in control, untreated GDT media (Fig. 4c). Although cells in rapamycin-treated full media show opposite regulations of mTORC1/AMPK compared to growing cells in GDT media, they were unable to elicit developmental gene induction. However, using GDT media, with only a slight reduction in nutrient/energy supply compared to full nutrient media, rapamycin was able to fully induce and replicate early developmental events for regulation of PKA, ACA, CAR1, csA, ERK1, and ERK2, as defined by starvation with DB (Fig. 4c). Under both conditions, the aggregation genes were similarly upregulated.

### Uncovering early regulated pathways for GDT

**Fig. 5:**
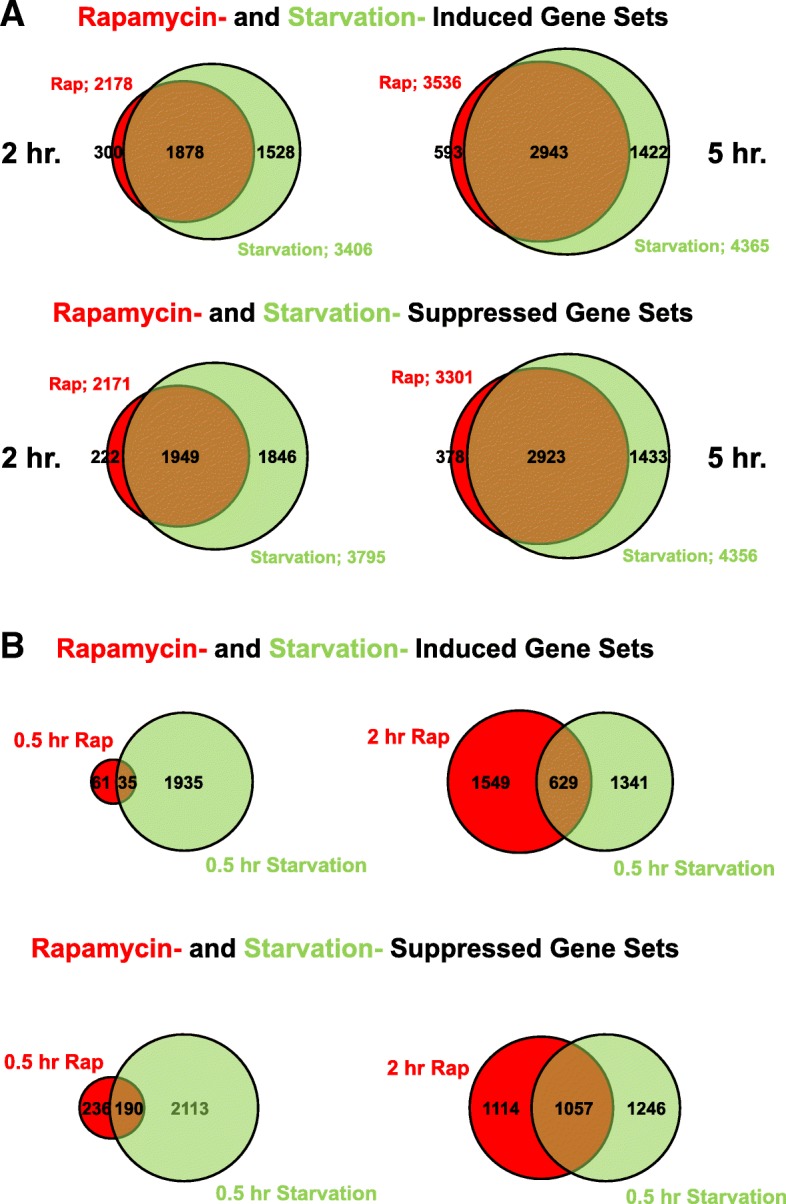
Comparative global gene expression changes by starvation and rapamycin-treated GDT media. Log-phase cells were adhered to cell well plates in GDT media. After 2 h, GDT media were replaced with DB or fresh GDT media with or without rapamycin. Cell samples were taken from controls and from the DB and rapamycin cells at varying times for RNA-seq. RNA-seq was in 3 replicates and up- and down-expressions determined as all significant differences (*q* < 0.1) comparing control values to developed values for DB or rapamycin cells after 30 min, 2 h, or 5 h. **a** Venn diagrams of induced/suppressed genes from starved or rapamycin-treated cells at times indicated, with total and overlapping gene numbers indicated and displayed proportionally. **b** Venn diagrams of induced/suppressed genes between 0.5 h of starvation and 0.5 or 2 h of rapamycin treatment, respectively, with total and overlapping gene numbers indicated, and displayed proportionally. Genes that overlap between 2 h of rapamycin treatment and 0.5 h of starvation are predicted to define classes of early regulated genes that are essential for growth-to-development transition (GDT), whereas gene classes regulated by starvation that do not overlap with rapamycin-treated cells are predicted to define non-essential classes (see Fig. 6)

Nutrient withdrawal induces development and, within 2 h, the up- and downregulation of > 7000 of genes ([43, 44]; Fig. 5a, Additional file 6: Table S3), > 50% of the entire *Dictyostelium* transcriptome. We reasoned that only a limited set of these genes may be connected directly to developmental dependency and that others may involve cellular response to the stress of nutrient removal. Using RNA-seq technology, we compared gene expression sets that change during development upon transfer from growth in GDT medium to DB with gene expression sets that change during development upon rapamycin treatment of cells growing in GDT media. We applied lfcShrink to estimate the log-fold change values and considered significant expression differences with *q* values < 0.1. We, thus, identified developmental gene changes, unrelated to nutrient withdrawal and starvation-associated stress. We note that only minimal differences in gene expression patterns are seen in full media control cells, treated with rapamycin (Additional file 6: Table S3).

As seen, ~ 3400 genes are induced and ~ 3800 genes suppressed at 2 h of starvation (Fig. 5a); these numbers increase to ~ 4500 in each group by 5 h. However, while nearly every rapamycin-induced or rapamycin-suppressed gene is similarly grouped with the corresponding starved gene class (Fig. 5a), the total number of rapamycin-regulated genes is strikingly fewer, by ~ 3000 at either 2 or 5 h (Fig. 5a; Additional file 6: Table S3).

Although an extended developmental delay for the rapamycin-treated cells could account for the vast differences in gene numbers, this is not the explanation. We considered if there were a significant overlap of late rapamycin and early starvation gene classes, comparing 5 h rapamycin-treated cells and 2 h starved cells. We show that ~ 2500 genes exhibit expression changes with 5 h rapamycin treatment, but not with 2 h rapamycin treatment. However, only ~ 15% of these 2500 later rapamycin-regulated genes are similarly grouped with 2 h starved cells (Additional file 7: Figure S4A), indicating that the 5 h rapamycin cells are temporally more advanced than the 2 h starved cells. Thus, we suggest that, although, rapamycin-induced development may be initially delayed in comparison with DB starvation (see Figs. 1e and 4b), during the 2–5-h developmental period, cells cultured under both rapamycin and starved conditions have initiated signal relay, streaming, and aggregation gene expression. When all times are considered, we suggest that there are > 2500 genes that exhibit expression changes upon 2 h starvation-induced development that is not similarly regulated by rapamycin treatment, indicating that these expression changes are not required to support development.

Conversely, development of 30 min rapamycin cells is delayed compared to 30 min starved cells (see Figs. 1e and 4b), and hence, their expression profiles show limited overlap (Fig. 5b). While the small group overlap may predict some essential regulatory genes, we wished to expand the analyses of gene sets where developmental dependency is linked to defined expression changes. We chose to compare 2 h rapamycin cells to 30 min starved cells (Fig. 5b). At 30 min starvation and 2 h rapamycin, 629 genes are induced and 1057 genes are suppressed. We believe that these genes represent a significant and most essential core for the early growth/development switch. Of the > 2500 genes regulated by starvation at 30 min but not by rapamycin at 2 h (Fig. 5b), ~ 85% group with 2 or 5 h starved cells (Additional file 7: Figure S4B), consistent with their regulations not essential for early developmental foundations.

### Gene networks and GDT

To first assess our hypothesis that the rapamycin experiments defined a more limited gene set for GDT than by starvation, we compared Gene Ontology annotations among the various groups, induced by rapamycin and starvation, suppressed by rapamycin and starvation, or unaffected by rapamycin (Table 1). First, for all classes, 40–50% of the genes are as yet unclassified, and an additional ~ 20% are involved in metabolic circuits (Additional file 8: Table S4). We thus focus on the non-metabolic, classifiable genes in each class.

**Table 1:**
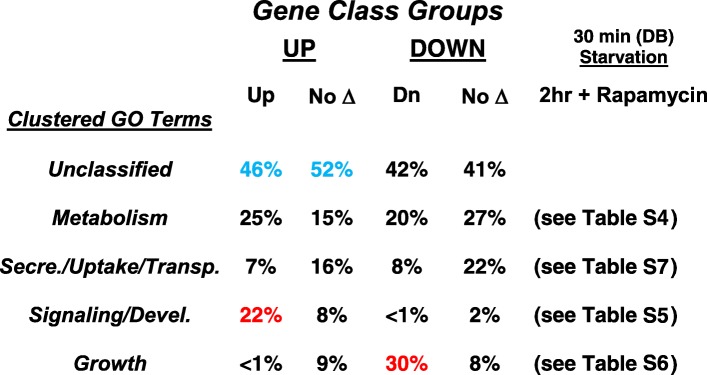
GO clustering of genes that are up- or downregulated at 30 min starvation, in group comparison with 2 h rapamycin treatment

WT cells were grown in GDT media and the culture divided. Rapamycin was added to 500 nM for one part and the other was transferred to DB. The analyses used RNA prepared from the growing cell controls, rapamycin cells at 2 h, and DB cells at 30 min, for RNA-seq. Experiments were conducted with 3 independent replicates. Thirteen thousand seven hundred twenty-nine gene numbers were recognized for each analysis

Genes with significant changes in expression compared to growth were re-grouped between the rapamycin and DB (starved) cultures (see Fig. 5b). Gene sets were analyzed for GO terms. GO terms could be placed into 4 overall clusters. 40 to 50% of the genes in each set could not be classified

**Fig. 6:**
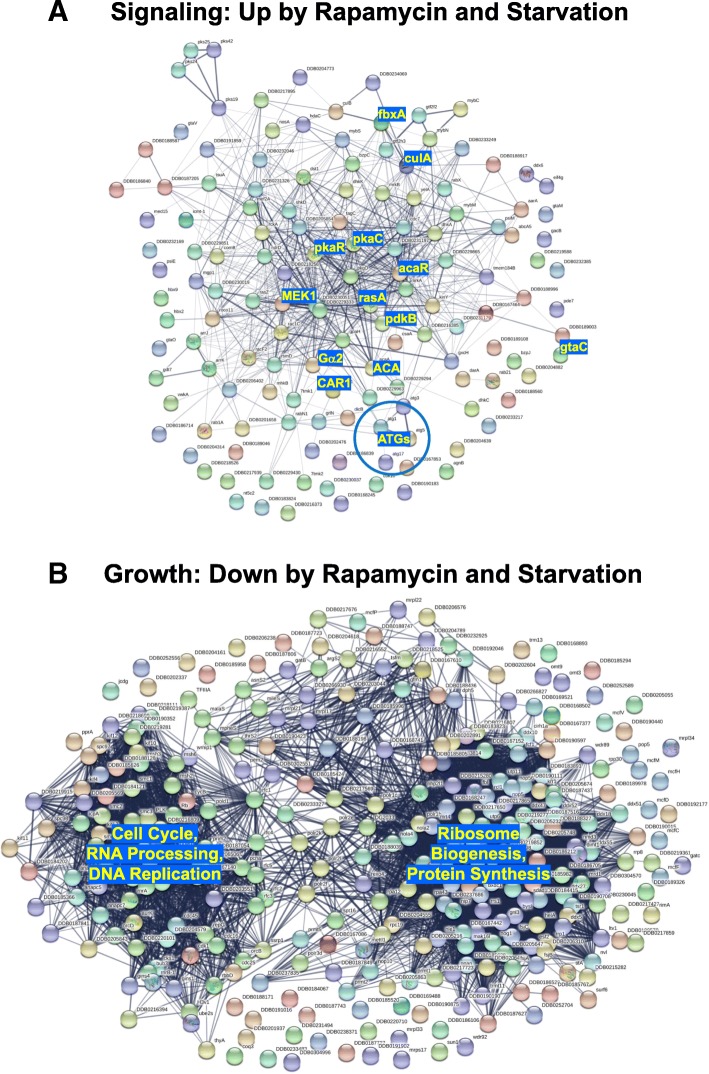
Gene Ontology network analysis of rapamycin/starvation regulation genes. **a** Approximately 135 genes are induced by both rapamycin and starvation with GO terms for developmental signaling. These were grouped for network association [45]. A major known cAMP signaling protein link involving cAMP synthesis (AcaR, ACA), cyclase regulation (PdkB, RasA), PKA (pPKAc, PKAr), cAMP receptor response (CAR1, Gα2), cAMP turnover (FbxA, CulA, MEK1), and TF target (GtaC) is indicated. ATGs group a class of genes involved in autophagy. Many genes are listed with dictyBase [46, 47] DDB0 genes names. These are clustered based upon their individual annotated properties. **b** Approximately 300 genes are suppressed by both rapamycin and starvation with GO terms for growth regulation. These were grouped for network association [45]. Two major clusters are noted, for cell division and protein synthesis

**Fig. 7:**
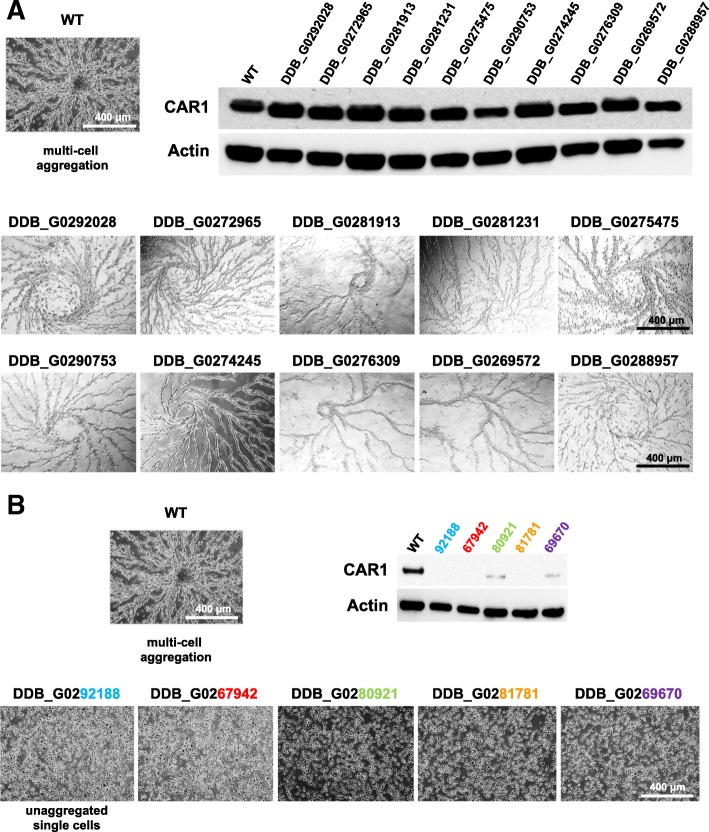
Assay for developmental abnormality to discover novel genes forming essential core for early development. **a** Normal early multi-cell development is observed for null cells from the unclassified starvation-alone induced gene group (see Fig. 5b). Mutant cells were plated and observed for multi-cell aggregation and induction of developmental marker-CAR1 expression after 5 h of starvation under developmental buffer. **b** Mutant cells from the unclassified rapamycin-induced class (see Fig. 5b) were unable to initiate multi-cell formation. Mutant cells were plated for multi-cell aggregation and assayed for induction of developmental marker-CAR1 expression after 5 h of starvation under developmental buffer. All lines remained as single cells and did not induce developmental gene expression

Red colors highlight the strong GO cluster bias of rapamycin-induced (up) genes for signaling (see Fig. 6a) and rapamycin-suppressed (down) genes for growth (see Fig. 6b). Blue colors highlight unclassified genes induced by rapamycin or by starvation alone (see Fig. 5b); genes were randomly selected from each class as a basis for gene function in control of developmental induction (see Fig. 7)

A most striking difference is seen comparing genes that are upregulated by rapamycin/starvation to those downregulated by rapamycin/starvation; 41% of the classified, upregulated genes (22% of total) are involved in the signaling paths (Table 1, Additional file 9: Table S5), with connection through an essential signaling network dependent upon CAR1/Gα2/ACA/PKA (Fig. 6a). Conversely, for the downregulated set, 52% (30% of total) are growth related (Table 1, Additional file 10: Table S6), either through cell cycle/DNA replication networks or protein synthesis (Fig. 6b). Not surprisingly, only a few developed, rapamycin- and starvation-activated genes show GO terms associated with growth.

For the genes not regulated by rapamycin, < 15% (< 10% of total) show a correlation to signaling/development or growth (Table 1), and potential network associations are very limited (Additional file 11: Figure S5A, B). A far greater number of genes in these sets (Table 1) involve pathways for secretion, uptake (e.g., endocytosis, pinocytosis), and intracellular transport and localization (Additional file 12: Table S7).

We conclude that rapamycin-induced development in GDT growth media defines a limited gene set essential for developmental induction. We suggest that a significant fraction of the ~ 300 unclassified genes that are upregulated by both rapamycin and starvation represent novel targets and pathway parts for developmental dependency. Since growth and development are competitive phases, the downregulation of ~ 300 growth-related genes (Table 1) may establish a permissive state for the response to developmental induction.

### Novel genes that regulate development

Regardless of gene set groups, ~ 50% of genes do not have classifiable orthologs in other systems (Table 1). We suggest that rapamycin-induced development, rather than starvation response, would define novel sets for gene mining. To this, we selected starvation-induced genes for mutation targeting from the unclassified list with and without grouping with the rapamycin-induced set. Genes with developmental induction of ~ 3-fold were chosen at random; 10 genes were selected from the starvation alone group (Table 1) and 5 genes from the rapamycin-overlap. Null cells were plated for development and also assayed for expression of developmental marker CAR1.

As seen in Fig. 7a, none of the null mutants from the selected starvation-alone gene set were developmentally compromised; all of them demonstrated multi-cell aggregation and expressed CAR1 to levels similar to that of WT cells. Thus, as hypothesized, we argue that genes that are not induced by rapamycin are less likely to play an essential role for early development. In contrast, development is significantly dependent on genes that are induced by rapamycin (Fig. 7b). None of the null mutant cells from the rapamycin class were able to aggregate but remained as single cells under standard starvation developmental conditions. In addition, expression of CAR1 was severely compromised in all 5 null cells. Although none of these 5 developmentally essential genes could be classified with functional homologs in other species, analyses with I-Tasser [48, 49] predicted significant GO and ligand binding functions for each, providing focus for potential developmental pathway linkages (Table 2).

**Table 2:** I-TASSER GO/functional predictions for unclassified, rapamycin-induced genes that regulate development (see Fig. 7b, Table 1)

Gene ID and protein size	Biological function	Molecular function	Cellular component	Ligand binding and homology domains
DDB_G0292188 750 amino acids	GO:0007166 Cell surface receptor signaling pathway	GO:0004888 Transmembrane signaling receptor activity	GO:0016021 Integral component of membrane	Glutamic acid; zinc von Willebrand factor (vWF) type A domain
DDB_G0267942 898 amino acids	GO:0033365 Protein localization to organelle	GO:0005515 Protein binding	GO:0031967 Organelle envelope	Zinc Gram domain
DDB_G0281781 620 amino acids	GO:0016477 Cell migration	GO:0004872 Signaling receptor activity	GO:0005924 Cell-substrate adherens junction	Glucose; calcium (None)
DDB_G0269670 568 amino acids	GO:0007018 Microtubule-based movement	GO:0032559 Adenyl ribonucleotide binding	GO:0030286 Dynein complex	Zinc; magnesium; iron C2 and WW domains
DDB_G0280921 1049 amino acids	GO:0030032 Lamellipodium assembly	GO:0005515 Protein binding	GO:0044424 Intracellular part	Zinc; magnesium; calcium Phox homology domain

Annotated protein sequences [46, 47] of characterized early Rapamycin upregulated genes (see Fig. 7b, Table 1) were analyzed by I-Tasser [48, 49]

Finally, we suggest that an effective parallel would exist for the downregulated gene classes. We anticipate that genes only regulated by starvation would generally not impact growth/development choice. Conversely, the downregulated rapamycin class may be closely coupled to growth. Although we have not yet studied these, many gene deletions may not be viable or may have severe growth defects.

## Discussion

*Dictyostelium* are professional phagocytes that grow in the wild as single cells, using bacteria as a food source. When nutrients are depleted, *Dictyostelium* switch from growth phase to development, leading to multi-cell aggregation. Nutrient depletion causes a rapid (~ 30 min) unloading of polysomes [50], from > 85 to < 40%, and expression changes to > 4000 genes [43, 44], ~ 35% of the entire *Dictyostelium* transcriptome. We wished to identify new gene sets and networks critical to the growth-to-development transition (GDT) and hypothesized that stress of standard nutrient depletion by washing cells into 10 mM phosphate buffer might affect many cellular processes, apart from developmental induction. Since mTORC1 and AMPK are poised at a nexus for nutrient sensing and cellular energy, we reasoned that their activity states might be determinant for *Dictyostelium* GDT.

Although mTORC1 was the central target for initial manipulation by rapamycin, reciprocal cross-talk with AMPK is essential. Indeed, we show that their two activities are inversely related and that each functionally inhibits the other. We suggest that phosphorylation of raptor by activated pAMPKα, as in other systems [10, 11], directly suppresses the activity of mTOR complex 1 in *Dictyostelium*. Activation of AMPK following mTORC1 inhibition is less direct but correlates with elevated AMP/ATP ratios. Metabolome data (unpublished) suggest that reduced glycolytic flux in rapamycin-treated cells may lead to increased AMP/ATP and consequently activated pAMPKα. Following starvation, where reduced amino acids and energy sources respectively suppress mTORC1 and activate AMPK, pathway cross-talk between these kinases stabilizes these activity changes. We propose that rapamycin treatment in GDT media is functionally equivalent to nutrient withdrawal, priming the initial trigger to inhibit mTORC1, which elevates AMP/ATP. pAMPKα is activated, which maintains mTORC1 suppression and promotes developmental cell fate switching, even in the presence of exogenous nutrients. Still, very highly enriched nutrient sources are able to override these subtle interregulations of mTORC1 and AMPK. We, thus, emphasize that cells can respond differently to subtle changes in extracellular nutrient levels and that nutrient stimulation may regulate pathways separate from mTORC1.

Standard axenic growth media for *Dictyostelium* include rich peptone and yeast extract sources, as well as glucose to > 80 mM; most amino acid concentrations are at > 5 mM. While we recognize the extreme physiological differences of mammalian cells and *Dictyostelium*, *Dictyostelium* full growth media has ~ 4–10-fold greater concentrations of glucose and essential amino acids compared to that for mammalian cell culture, and in such highly rich media, rapamycin has only limited effect to suppress the growth of *Dictyostelium*. Since rapamycin does not inhibit mTORC1 phosphorylation of all nutrient-regulated substrates in rich media in other systems [30, 31], we systematically analyzed amino acid and cellular energy effects on mTORC1/AMPK in *Dictyostelium* for growth and developmental response to rapamycin. We then narrowly defined nutrient conditions [e.g., an essential 30% reduction in glucose concentration (Fig. 3d)] that permitted rapid axenic growth, but also sensitivity to rapamycin-induced development.

By extensive comparison of the transcriptomes of starvation-induced cells and rapamycin-treated cells, we propose that global upregulation of < 700 genes, and perhaps < 500, during very early development may mark a defining interactive network for GDT.

The YakA kinase is an essential activator for PKAc mRNA expression in *Dictyostelium* [35, 36] and an early effector downstream of mTORC1 inhibition. PKA plays a critical role in *Dictyostelium* development [51, 52]. Cells deficient in PKA are unable to induce development, whereas cells with unregulated activated PKA will develop precociously and bypass many developmental arrest mutations [53, 54]; neither *pkaC*-null cells nor *yakA*-null cells are able to develop. We have shown rapid phospho-activation of YakA during both starvation- and rapamycin-induced development, indicating an early mode for mTORC1 regulation of PKA. We suggest that mTORC1 inhibition promotes YakA activation and, thus, PKA transcription. Coordinated with PKA gene activation, we show starvation- and rapamycin-induced upregulation of ~ 135 transcripts, whose encoded proteins form a cAMP-centric signaling network (Fig. 6a), including both early developmental cyclases for cAMP synthesis (AcaR, ACA), the receptor sensor complex (CAR1, Gα2), cAMP turn-over components (MEK1, FbxA, CulA), cyclase regulators (RasA, PdkB), PKA complex members (PkaC and PkaR), and finally the PKA-regulated transcription factor GtaC, which is itself a primary activator of CAR1 and other genes in the network pathway [55]. Since these signaling pathway genes represent ~ 50% of all classified, rapamycin upregulated genes, we speculate that an additional 100–150 genes that are not yet classified to the genetic relationship may also participate in early developmental signaling, and represent new modes for study.

To the converse, genes that are downregulated upon rapamycin treatment form two large (~ 300) gene networks associated with growth processes of cell cycle and protein synthetic machinery (Fig. 6b). This sets a curious analogy to *Saccharomyces*, where the activation of YAK1-related kinase is also connected to the suppression of ribosomal protein gene transcription, via mTORC1 and PKA [56]. In both *Saccharomyces* and *Dictyostelium*, inactivated mTORC1 leads to activated YAK1/YakA, which represses ribosomal protein expression. But the actual mechanistic pathways are distinct. In yeast, activated PKA is an upstream repressor of YAK1 [56], whereas in *Dictyostelium*, PKA lies downstream of YakA, in a PKA activation pathway [35, 36]. Following nutrient withdrawal in yeast, YAK1 undergoes cytosol-to-nucleus translocation upon phosphorylation [56]. Although we similarly observe rapid phosphorylation of YakA in *Dictyostelium* with nutrient withdrawal, YakA does not translocate but remains cytosolic (unpublished).

Our data also address the connection of mTORC1 and autophagy in *Dictyostelium* [57]. As nutrients become limiting, excess macromolecules are degraded by autophagic processes to maintain essential cellular functions [58]. Active mTORC1 suppresses autophagy, but rapamycin inhibition of mTORC1 is often sufficient to activate autophagy, even in the presence of nutrients [2]. *Dictyostelium* which have been starved for nutrients will induce autophagy, which targets ribosomal machinery and other components for degradation. This catabolic process is essential to replenish needed precursors for cellular reprogramming, and *Dictyostelium* that are unable to undergo autophagy are blocked in development [59]. Our modified medium conditions support rapamycin-induced development as well as expression of ATG-class genes required for autophagy (Fig. 6a).

Active/inactive mTORC1 and nutrient balance in control of cell fate determination may be a more widely applied paradigm. In the immune system, this is well described, where the relative activity of mTORC1 can be a determining factor for the differentiation of effector, memory, or regulatory T cells [14, 15, 17] and reflect the cellular metabolic state and perhaps environmental nutrients [14, 16–18].

We have established conditions that alter cell fate, albeit a growth-to-development transition, upon direct manipulation of mTORC1/AMPK activities. This approach obviates the requirement for full media withdrawal and a shift to simple low ionic strength buffer, with an accompanying starvation-associated stress. To this, we argue that most of the 4000 gene expression changes observed upon starvation for 30 min (Fig. 6b) are not related to developmental necessity. Many fewer genes related to the signaling pathways are upregulated by starvation alone, as compared with rapamycin induction (Table 1). Likewise, most starvation-specific, downregulated genes are not coupled to paths for cell growth.

We, thus, argue that perhaps < 500 genes that are upregulated within the first 30 min of starvation-induced development play a critical developmental signaling role. Although many of these are known and studied, we have identified whole new gene sets that have no known metazoan homolog but may integrate essential novel signaling motifs. To support this directly, we show that 5 randomly selected genes from the unclassified, rapamycin-induced set had a perfect correlation to developmental dependency, whereas none from the starvation-induced set impacted development in a significant manner.

## Conclusions

This study indicates that rapamycin-targeted inactivation of mTORC1 with reciprocal activation of AMPK, in the absence of nutrient withdrawal, is sufficient to effect a growth-to-developmental fate switch to induce multi-cell development of *Dictyostelium*. Using an RNA-sequencing approach, we identified mTORC1/AMPK-regulated transcriptional networks and associated signaling pathways that are essential for early developmental induction but are regulated independently of nutrient withdrawal. We then investigated genes with unclassifiable GO and ortholog terminologies and showed that the rapamycin-induced expression group can be applied for novel gene discovery in pathways essential for early developmental induction.

## Materials and methods

### Cell culture and cell lines

*Dictyostelium* AX3 strain [46, 47], *rheb*^−^ KO, *tsc2*^−^ KO, *fkbp*12^−^ KO, *lst8*^−^ KO, *RAPT*^OE^, and *YakA*^−^/YakA^−GFP^ cells were grown axenically in HL5 medium with glucose (ForMedium #HLG0102) containing 100 μg/ml of ampicillin and 100 μg/ml streptomycin, at 22 °C in suspension culture, shaking at ~ 180 rpm, to a density of 1–1.5 × 10^6^ cells/ml. *YakA*^−^/YakA^−GFP^ cells [46, 47] were grown under selection in 5 μg/ml blasticidin (Invivogen # ant-bl) and 50 μg/ml G418 (Gold Biotechnology # G-418-5). *rheb*^−^ KO, *tsc2*^−^ KO, *fkbp*12^−^ KO, and *lst8*^−^ KO [24] cells were maintained under selection in 5 μg/ml blasticidin, as were mutants in DDB_G0292028 (GWDI_354_B_11), DDB_G0272965 (GWDI_188_C_7), DDB_G0281913 (GWDI_48_H_4), DDB_G0281231 (GWDI_421_G_11), DDB_G0275475 (GWDI_424_H_7), DDB_G0290753 (GWDI_188_B_8), DDB_G0274245 (GWDI_424_B_9), DDB_G0276309 (GWDI_423_E_8), DDB_G0269572 (GWDI_165_C_11), DDB_G0288957 (GWDI_526_D_1), DDB_G0292188 (GWDI_526_A2), DDB_G0280921 (GWDI_491_A4), DDB_G0269670 (GWDI_189_C8), DDB_G0267942 (GWDI_483_D2), and DDB_G0281781 (GWDI_477_H6) [46, 47].

Rapamycin (Sigma-Aldrich # 37094) was used at 500 nM, as previously established [24, 26]. pAICAR (phospho 5-aminoimidazole-4-carboxamide ribonucleotide; Sigma-Aldrich # A1393) was used at 1 mM, based on previous dose studies [37] and optimization for pAMPK activation. Eighty millimolar 2-deoxy-d-glucose (2-DG; Sigma-Aldrich # D6134), at 1:1 with media glucose, was sufficient to rapidly (in < 10 min) reduce cellular levels of ATP to 0.1× and, thus, activate AMPK. Similar pAMPK activation was seen to 320 mM 2-DG, whereas no pAMPK activation was detected at 40 mM 2-DG in the presence of 80 mM glucose. Dorsomorphin (TOCRIS # 3093) was used at 40 μM. Forty micromolar dorsomorphin did not alter growth rate over 48 h, whereas 120 μM dorsomorphin caused lethality overnight.

### Cell growth rates assay

To measure the cell growth rates, we diluted cells from log-phase growth (1–1.5 × 10^6^ cells/ml) into the fresh growth media at 0.1 × 10^6^ cells/ml and incubated at 22 °C with constant shaking at ~ 180 rpm. Cell growth was monitored by counting cells using a cell counter machine [Cellometer Vision-Nexcelom Bioscience] at regular time intervals over several days. Cell growth assays were performed in triplicate sets in each independent experiment (*N* = 3).

### Immunoblotting

For immunoblotting, whole cell lysates were prepared in Laemmli lysis buffer (Bio-Rad # 161-0747) containing 2.5% of β-mercaptoethanol and boiled for 10 min at 95 °C. Phosphorylation status of S6K, 4EBP1, AMPKα, and ERK1/2 proteins was monitored in whole cell lysates by immunoblotting following gel electrophoresis (Bio-Rad, 4–20% gradients Tris-glycine gels), with antibodies against human pSGK1^S422^ for S6K (at a dilution of 1:1000; Abcam # ab55281), p4EBP1^T70^ (1:500; Cell Signaling # 9455), pAMPKα^T172^ (1:2000; Cell Signaling # 2535), and pERK^T202/Y204^ (1:1000; Cell Signaling # 9101) [24, 60]. For 4EBP1 proteins, gels were transferred onto 0.2-μm PVDF membranes, followed by blocking with non-fat dry milk (Thermo-Scientific # 37530) for 1 h at room temperature. YakA phosphorylation was studied in YakA^−^/YakA^−GFP^ cell lysates, using antibodies against GFP (Cell Signaling # 2956) and phosphoTyrosine (pY) residue (BD Transduction laboratories # PY20).

Expression levels of pS6K, p4EBP1, and pY were detected using the Femto level ECL substrate (Thermo-Scientific # 34096). The cellular development kinetics was monitored by immunoblotting with antibodies against *Dictyostelium* PKAr (DSHB # 112-315-26), csA (DSHB # 12-120-94/6), ACA (at a dilution of 1:5000 dilution [61]), CAR1 (at a dilution of 1:5000; [62]), and actin proteins (Santa Cruz Biotechnology; Sc-1616 HRP). For CAR1 proteins, cells after lysis with Laemmli lysis buffer containing 2.5% of β-mercaptoethanol were not followed with boiling step.

### Quantification of AMP and ATP levels

To quantify the AMP and ATP content in cells, we lysed 1 × 10^7^ pelleted cells by freeze-thaw in liquid nitrogen and subsequent shift to − 80 °C for overnight. Lysed cell pellets were re-suspended in 50 μl of water, centrifuged at 10,000 rpm for 10 min at 4 °C, and supernatants assayed. AMP levels were measured by luminescence detection using the AMP-Glo assay kit (Promega # V5011), following the manufacturer’s instructions. Ten microliters of supernatant was mixed with 10.0 μl of AMP-glow reagent-I for 1 min and incubated at 22 °C for 1 h. Twenty microliters of AMP detection solution was added, mixed, incubated at 22 °C for 1 h, and luminescence measured as described. ATP levels were measured using the ATPlite luminescence assay system (PerkinElmer # 6016943), following the manufacturer’s instructions. Ten microliters of supernatant was mixed with 10.0 μl of ATPlite substrate solution in the dark for 10 min and luminescence measured. We normalized the AMP and ATP levels with protein concentrations in each sample. Two microliters of supernatant was mixed with 100 μl of Bradford protein assay reagent (Bio-Rad # 5000201), incubated for 15 min, and concentration measured by absorbance at 595 nm.

### Quantitative phospho-proteome analysis by mass spectrometry

Samples for phospho-proteome analysis were compared from cells in growth media or following 15 min starvation in DB. To prepare the samples, cells were lysed in RIPA buffer (ThermoFisher # 89900) containing Phosphatase Inhibitor Cocktail (PhosSTOP at 1×; Roche # 04906837001) and Protease Inhibitor Cocktail (at 1×; Roche # 04639159001), followed by immediate freezing in liquid nitrogen. Samples were labeled with TMT, phosphopeptide-enriched using TiO2, and processed by MyOmicsDx, Inc. (Towson, MD) for mass spectrometry analyses.

### *Dictyostelium* development

To examine *Dictyostelium* multi-cellular development in starvation condition, log-phase growth cells (1–1.5 × 10^6^ cells/ml) were adhered in 6-well plates (3.5 × 10^6^ cells/well or 0.4 × 10^6^ cells/cm^2^), replenished with fresh full growth media or GDT growth media and incubated for 2 h. Cells were then washed twice with developmental buffer (DB; 5 mM Na_2_HPO_4_, 5 mM NaH_2_PO_4_, 0.2 mM CaCl_2_, 2 mM MgCl_2_, adjust to pH 6.6) and then replenished with 2.0 ml of DB. Developmental progress was then monitored at regular intervals.

To examine the growth-to-development transition induced by rapamycin without nutrient withdrawal, cells adhered in 6-well plates were washed twice with GDT growth media, replenished with 2 ml of GDT media [50% Glc^[-]^ (HL5 media without glucose, Formedium # HLB0102), 27 mM glucose, 1 mM MgCl_2_, 0.5 mM CaCl_2_, adjust to pH 6.6] media and incubated at 22 °C for 2 h, followed with rapamycin (Sigma-Aldrich # 37094) treatment to the GDT-media. Developmental progress was then monitored at regular intervals.

### RNA extraction and RNA sequencing

To determine the essential core genes regulating growth-to-development transition (GDT), RNA sequencing techniques were adapted. Total RNA from the growing or developing cells were isolated using the RNAeasy mini preparation kit (Qiagen # 74104) and following the manufacturer’s protocol. RNA integrity was checked by running a denaturing MOPS-formaldehyde gel and with the Agilent bioanalyzer. Four micrograms of total RNA was used for poly(A) enrichment and followed with mRNA sequencing library preparations using the Truseq mRNA library preparation kit (Illumina # RS-122-2102). Fifty base pair single-end were sequenced on the Illumina HiSeq 2500. Adapters were trimmed using cutadapt v1.13 [63], with 3′ quality trimming of 20, minimum read length of 25, and default parameters otherwise. The filtered and trimmed reads were aligned to the ENSEMBL *Dictyostelium discoideum* reference genome with HISAT v2.1.0 [64] and default parameters. Gene-level counts were aggregated from the aligned reads using Subread featureCounts v1.6.0 [65, 66] and default parameters, and the ENSEMBL cDNA and ncRNA annotation datasets. Count data were analyzed for differential expression between conditions using DESeq2 v1.18.1 [67] in R v3.4.1. Moderated log-fold change estimates were generated using the “lfcShrink” method as recommended by the DESeq2 development team. RNA-seq data can be accessed with GEO [68, 69] repository link: https://www.ncbi.nlm.nih.gov/geo/query/acc.cgi?acc=GSE123599.

### Gene Ontology and gene network

GO terms of individual genes were analyzed using DAVID 6.8 Version [70] (https://david.ncifcrf.gov/) and classified on the basis of biological function under category GOTERM_BP_ALL. Gene network models of the interested genes were predicted using the STRING 10.5 Version [45] (https://string-db.org/) with an interaction score of 4.5 confidence. Some individual genes were further analyzed with I-Tasser [48, 49].

## Additional files

Additional file 1: **Figure S1.** Regulation of relative levels of ATP/AMP through mTORC1. Quantification of relative AMP/ATP ratios upon nutrient withdrawal (DB) or rapamycin treatment in full growth media (Med+Rap) in shaking culture. At times indicated, 1 × 10^7^ cells were pelleted and lysed by freeze-thaw. The AMP and ATP levels were measured separately, and values represent ratio changes as mean ± standard error. Results are from three independent experiments, with triplicates used for each independent set of experiments. (PDF 38 kb)

Additional file 2: **Figure S2.** Differential effects of rapamycin on growth regulation of cells lacking regulators of mTORC1. Relative growth rates of various cell lines treated with 500 mM rapamycin in comparison with the same, untreated cell population. Lst8 and Rheb are positive regulators of mTORC1, and cells lacking either are more sensitive to rapamycin inhibition than WT. TSC2 is a negative regulator of mTORC1, and cells lacking TSC2 are less sensitive to rapamycin inhibition than WT. FKBP12 is an essential regulator of rapamycin, and cells lacking FKBP12 are insensitive to rapamycin. Rapamycin inhibits growth by disrupting Raptor interaction with mTOR, and cells that overexpress Raptor are less sensitive to rapamycin than WT. Each of the experimental cell lines shows more minimal (< 20%) growth differences to WT in the absence of rapamycin. (PDF 46 kb)

Additional file 3: **Table S1.** Amino acid-energy state regulation of mTORC1/AMPK. (DOCX 21 kb)

Additional file 4: **Table S2.** Rapamycin induces the YakA/PKA/ACA/CAR1 network. (DOCX 30 kb)

Additional file 5: **Figure S3.** Rapid phospho-proteome changes in YakA upon starvation. Relative abundance ratio of the STLYTpYIQSR peptide (site probability > 0.99) within the activation loop of YakA (see Fig. 4b) during growth in GDT media and following 15 min starvation in DB, as analyzed from three independent preparations. (PDF 146 kb)

Additional file 6: **Table S3.** Transcriptome changes during developmental induction. (DOCX 26 kb)

Additional file 7: **Figure S4.** Venn diagrams of differentially expressed genes between starvation and rapamycin-treated GDT media. A. Venn diagram of differentially regulated genes from 2 to 5 h of rapamycin treatment in GDT media with 2 h of starvation, with percent overlap indicated and displayed proportionally. B. Venn diagram of differentially regulated genes from starvation alone at 0.5 h with starvation at 2 through 5 h, with percent overlap indicated and displayed proportionally. (PDF 49 kb)

Additional file 8: **Table S4.** Metabolism group. (DOCX 18 kb)

Additional file 9: **Table S5.** Signaling group. (DOCX 15 kb)

Additional file 10: **Table S6.** Growth group. (DOCX 16 kb)

Additional file 11: **Figure S5.** Gene Ontology network analysis of genes regulated by starvation but not by rapamycin. A. Approximately 110 genes are induced and ~ 25 genes suppressed by starvation and not rapamycin with GO terms for developmental signaling. These were grouped for network association [45], with only minimal interactions seen. B. Approximately 120 genes are induced and 100 genes suppressed by starvation and not rapamycin with GO terms for growth. These were grouped for network association [45]. (PDF 762 kb)

Additional file 12 **Table S7.** Secretion/uptake/transport group. (DOCX 17 kb)

## Data Availability

RNA-seq data can be accessed with GEO [68, 69] repository link: https://www.ncbi.nlm.nih.gov/geo/query/acc.cgi?acc=GSE123599 [71]. Cell lines and vectors are available or accessed at dictyBase (http://dictybase.org).
