# Supplementary figures and images for: mTORC1/AMPK responses define a core gene set for developmental cell fate switching
Source: BMC Biol. 2019 Jul 18;17:58. doi: 10.1186/s12915-019-0673-1 (PMC6637605; doi:10.1186/s12915-019-0673-1)

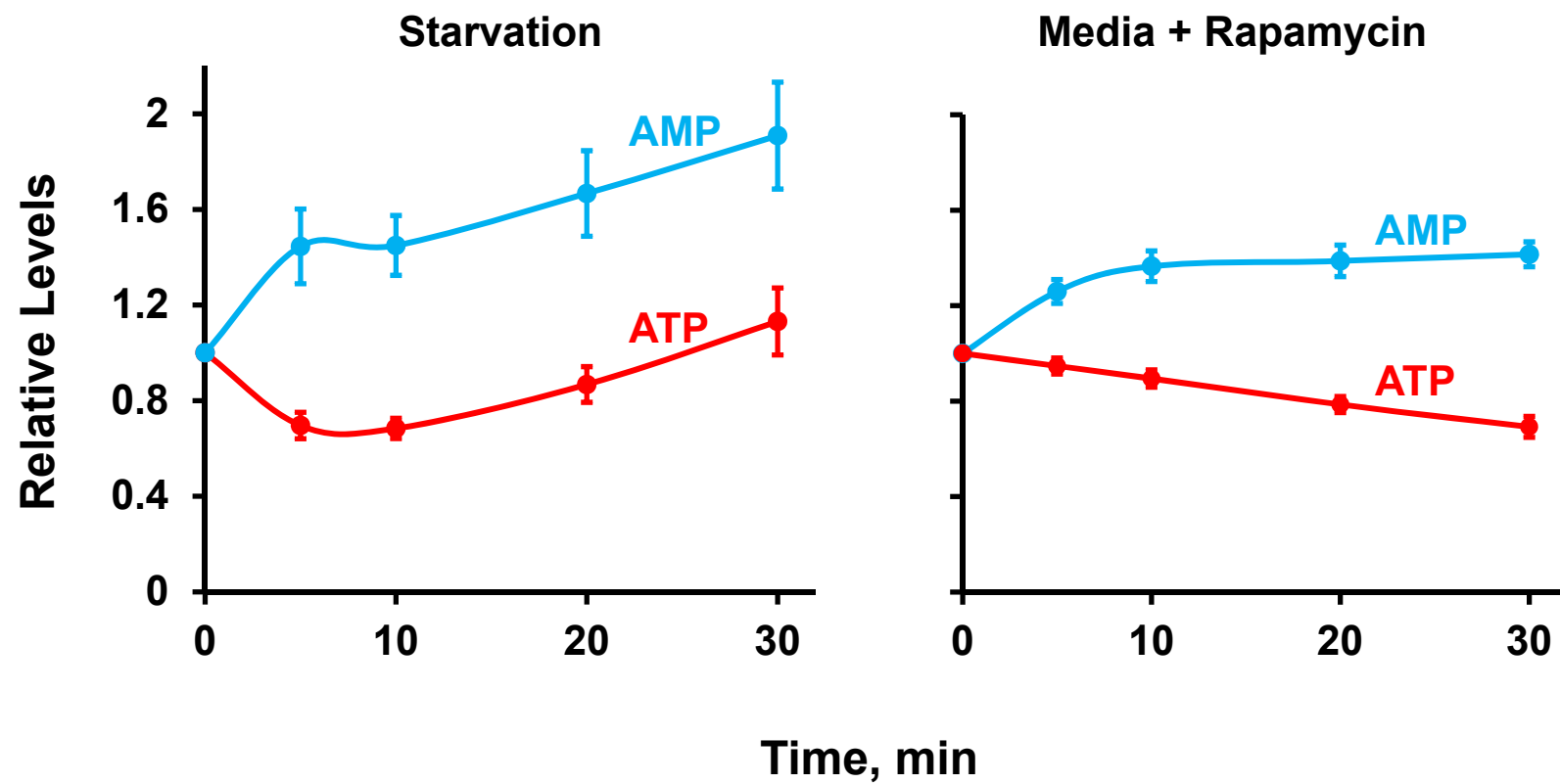

Supplementary Figure S1

Supplement: Supplementary file 1 — Figure S1. Regulation of relative levels of ATP/AMP through mTORC1. Quantification of relative AMP/ATP ratios upon nutrient withdrawal (DB) or rapamycin treatment in full growth media (Med+Rap) in shaking culture. At times indicated, 1 × 10^7^ cells were pelleted and lysed by freeze-thaw. The AMP and ATP levels were measured separately, and values represent ratio changes as mean ± standard error. Results are from three independent experiments, with triplicates used for each independent set of experiments. (PDF 38 kb) [file 12915_2019_673_MOESM1_ESM.pdf]

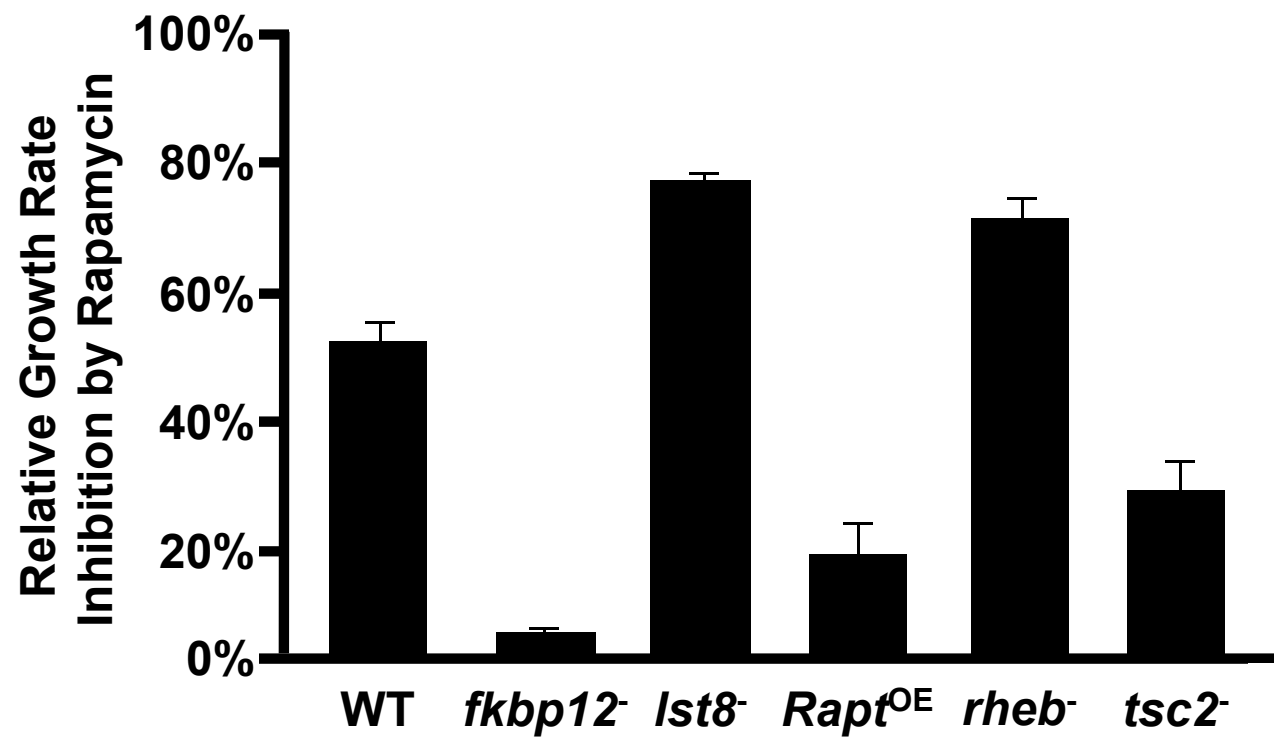

Supplementary Figure S2

Supplement: Supplementary file 2 — Figure S2. Differential effects of rapamycin on growth regulation of cells lacking regulators of mTORC1. Relative growth rates of various cell lines treated with 500 mM rapamycin in comparison with the same, untreated cell population. Lst8 and Rheb are positive regulators of mTORC1, and cells lacking either are more sensitive to rapamycin inhibition than WT. TSC2 is a negative regulator of mTORC1, and cells lacking TSC2 are less sensitive to rapamycin inhibition than WT. FKBP12 is an essential regulator of rapamycin, and cells lacking FKBP12 are insensitive to rapamycin. Rapamycin inhibits growth by disrupting Raptor interaction with mTOR, and cells that overexpress Raptor are less sensitive to rapamycin than WT. Each of the experimental cell lines shows more minimal (< 20%) growth differences to WT in the absence of rapamycin. (PDF 46 kb) [file 12915_2019_673_MOESM2_ESM.pdf]

**A.**

Rap, from 2-5 hrs.  
(15% overlap)

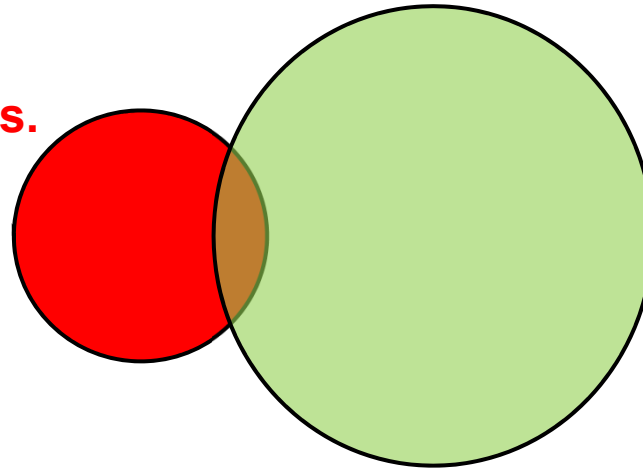

2 hr Starvation

**B.**

30 min. starve, not rap  
(85% overlap)

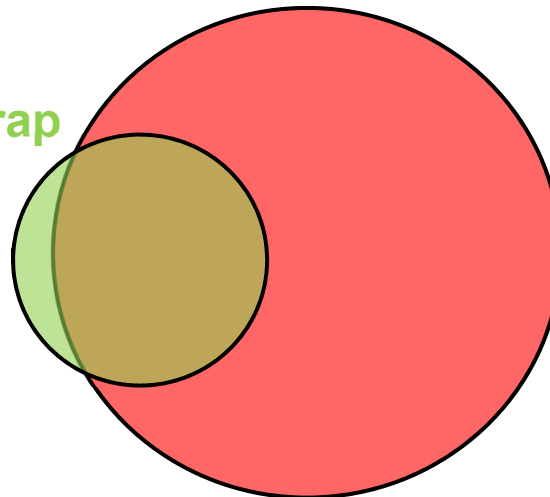

2/5 hr. Starvation

Supplement: Supplementary file 7 — Figure S4. Venn diagrams of differentially expressed genes between starvation and rapamycin-treated GDT media. A. Venn diagram of differentially regulated genes from 2 to 5 h of rapamycin treatment in GDT media with 2 h of starvation, with percent overlap indicated and displayed proportionally. B. Venn diagram of differentially regulated genes from starvation alone at 0.5 h with starvation at 2 through 5 h, with percent overlap indicated and displayed proportionally. (PDF 49 kb) [file 12915_2019_673_MOESM7_ESM.pdf]
