# Supplementary material for: mTORC1/AMPK responses define a core gene set for developmental cell fate switching
Source: BMC Biol. 2019 Jul 18;17:58. doi: 10.1186/s12915-019-0673-1 (PMC6637605; doi:10.1186/s12915-019-0673-1)
Supplement: Supplementary file 3 — Table S1. Amino acid-energy state regulation of mTORC1/AMPK. (DOCX 21 kb) [file 12915_2019_673_MOESM3_ESM.docx]

**Table S1**

**No**

**His**

**Thr
Iso**

**Phe**

**Gly**

**Met**

**Tyr**

**Asn**

**Yes**

**Glucose**

**Cys**

**Glu**

**Trp**

**Leu**

**Arg**

**Lys**

**Pro**

**Val**

Growing WT cells were transferred to DB starvation buffer, de-activating mTORC1 and inducing AMPK. At 30 min, the various nutrients above were individually added to the DB and changes in activation state of mTORC1 and AMPK were monitored after 15 min. Amino acids were added at 3-10 mM. Glucose was to 80 mM. Amino acids Asn, Asp, Gln, Tyr, Ser, and Ala are non-essential.

**Amino Acid-Energy State Regulation of mTORC1/AMPK**
