# Supplementary material for: mTORC1/AMPK responses define a core gene set for developmental cell fate switching
Source: BMC Biol. 2019 Jul 18;17:58. doi: 10.1186/s12915-019-0673-1 (PMC6637605; doi:10.1186/s12915-019-0673-1)
Supplement: Supplementary file 4 — Table S2. Rapamycin induces the YakA/PKA/ACA/CAR1 network. (DOCX 30 kb) [file 12915_2019_673_MOESM4_ESM.docx]

**Table S2**

**Rapamycin Induces the YakA/PKA/ACA/CAR1 Network**

**2 hr Expression (RNA-seq) Relative to Growth**

***PufA***

***YakA***

***PKAc***

***PKAr***

***ACA***

***CAR1***

**Growth Media**

**Treatment**

**D3T**

**+Rap**

**1.0**

**1.0**

**1.0**

**1.0**

**1.0**

**1.0**

**Bact.**

**Dev.**

**0.5**

**2.6**

**3.0**

**6.0**

**10.0**

**6.2**

**GDT**

**DB**

**0.5**

**2.6**

**3.9**

**6.3**

**13.8**

**11.6**

**GDT**

**+Rap**

**0.8**

**1.6**

**2.2**

**3.7**

**4.1**

**4.9**

**GENE**

WT cells were grown in one of two media preparations. One culture was grown in full nutrient media D3T; rapamycin was added to 500 nM. One culture was grown in GDT media and the culture divided. Rapamycin was added to 500 nM to one part. The remaining GDT cells were transferred to DB starvation buffer. RNA was prepared from the respective growing cell controls and from the treated cultures after 2 hr, as noted above, and analyzed by RNA-seq. Relative gene expression to growth is listed. Experiments were conducted with 3 independent replicates (see Table S III). Bacteria/Development data are from Parikh *et al*. (2010) and Rosengarten *et al*. (2015).
