# Supplementary material for: mTORC1/AMPK responses define a core gene set for developmental cell fate switching
Source: BMC Biol. 2019 Jul 18;17:58. doi: 10.1186/s12915-019-0673-1 (PMC6637605; doi:10.1186/s12915-019-0673-1)
Supplement: Supplementary file 5 — Figure S3. Rapid phospho-proteome changes in YakA upon starvation. Relative abundance ratio of the STLYTpYIQSR peptide (site probability > 0.99) within the activation loop of YakA (see Fig. 4b) during growth in GDT media and following 15 min starvation in DB, as analyzed from three independent preparations. (PDF 146 kb) [file 12915_2019_673_MOESM5_ESM.pdf]

Peptide Sequence STLYTYIQSR	Site Probability Y6(Phospho): 99.99%
Quality q-value	0.00275822

GDT Media	DB 15 min
**Abundance Ratio:** (F1, 128C) / (F1, 128C) [Summary of 3 replicates]	**Abundance Ratio:** (F1, 129C) / (F1, 128C) [Summary of 3 replicates]
**1**	**1.569**

Conditions	Labeling Scheme
GDT [0 min]	128C
Starvation [15 min]	129C

Supplementary Figure S3
