# Supplementary material for: mTORC1/AMPK responses define a core gene set for developmental cell fate switching
Source: BMC Biol. 2019 Jul 18;17:58. doi: 10.1186/s12915-019-0673-1 (PMC6637605; doi:10.1186/s12915-019-0673-1)
Supplement: Supplementary file 6 — Table S3. Transcriptome changes during developmental induction. (DOCX 26 kb) [file 12915_2019_673_MOESM6_ESM.docx]

**GDT**

**DB**

**3406**

**3795**

**GDT**

**+Rap**

**2178**

**2171**

**Growth Media**

**Treatment**

**D3T**

**+Rap**

**31**

**44**

***Up Regulation***

***Down Regulation***

**2 hr Gene Expression Changes Relative to Growth**

**Table S3**

**Transcriptome Changes During Developmental Induction**

WT cells were grown in one of two media preparations. One culture was grown in full nutrient media D3T; rapamycin was added to 500 nM. One culture was grown in GDT media and the culture divided. Rapamycin was added to 500 nM to one part. The remaining GDT cells were transferred to DB starvation buffer. RNA was prepared from the respective growing cell controls and from the treated cultures after 2 hr, as noted above, and analyzed by RNA-seq.

Numbers of genes with significant changes in expression compared to growth are listed for the 3 cultures. Experiments were conducted with 3 independent replicates. 13729 gene numbers were recognized for each analysis.
