# Supplementary material for: mTORC1/AMPK responses define a core gene set for developmental cell fate switching
Source: BMC Biol. 2019 Jul 18;17:58. doi: 10.1186/s12915-019-0673-1 (PMC6637605; doi:10.1186/s12915-019-0673-1)
Supplement: Supplementary file 8 — Table S4. Metabolism group. (DOCX 18 kb) [file 12915_2019_673_MOESM8_ESM.docx]

**Table S5**

**Metabolism Group**

**157 genes - up with rapamycin/starvation (catabolic bias)**

| **GO Term** | **Number of Genes/Count** | **P-Value** |
| --- | --- | --- |
| Metabolic process | 121 | 1.70E-24 |
| Macromolecule metabolic process | 87 | 1.10E-11 |
| Macromolecule catabolic process | 44 | 1.80E-25 |
| Macromolecule modification | 41 | 5.20E-07 |
| Protein catabolic process | 39 | 4.50E-27 |
| Regulation of protein catabolic process | 8 | 3.00E-07 |
| Proteasomal protein catabolic process | 21 | 1.80E-15 |
| Carbohydrate metabolic process  gluconeogenesis, amino acid-sugar,  glycosylation, oligosaccharide | 10 | 4.30E-02 |
| Carbohydrate derivative metabolic process | 12 | 4.40E-02 |
| Hexose metabolic process | 4 | 2.70E-02 |
| Amino acid-sugar metabolic process | 3 | 3.70E-02 |
| Organonitrogen catabolic process | 8 | 1.00E-02 |
| mRNA catabolic process | 3 | 7.10E-02 |

**211 genes - down with rapamycin/starvation (anabolic bias)**

| **GO Term** | **Number of Genes/Count** | **P-Value** |
| --- | --- | --- |
| Metabolic process | 199 | 1.70E-47 |
| Macromolecule metabolic process | 115 | 2.70E-07 |
| Macromolecule modification | 56 | 1.40E-06 |
| Protein metabolic process | 54 | 5.50E-02 |
| Carbohydrate derivative metabolic process | 12 | 5.60E-02 |
| Nucleic acid metabolic process | 68 | 8.50E-08 |
| RNA metabolic process | 55 | 1.50E-06 |
| Cellular nitrogen biosynthesis process | 104 | 2.30E-14 |
| Nucleoside phosphate metabolic process | 13 | 1.10E-02 |
| Organonitrogen compound catabolic process | 46 | 1.10E-05 |
| small molecule metabolic process | 46 | 6.80E-09 |
| Co-factor biosynthetic process | 19 | 8.50E-07 |

**202 genes - up with starvation only (catabolic bias)**

| **GO Term** | **Number of Genes/Count** | **P-Value** |
| --- | --- | --- |
| Metabolic process | 152 | 2.10E-25 |
| Macromolecule metabolic process | 93 | 4.20E-06 |
| Macromolecule catabolic process | 18 | 3.40E-03 |
| Macromolecule modification | 38 | 3.80E-03 |
| Protein catabolic process | 12 | 2.00E-02 |
| Proteasomal protein catabolic process | 9 | 3.60E-03 |
| Carbohydrate metabolic process | 14 | 7.40E-03 |
| Carbohydrate derivative metabolic process | 15 | 3.00E-02 |
| Organonitrogen compound catabolic process | 8 | 3.70E-02 |
| Lipid metabolic Process | 24 | 5.80E-06 |
| Phospholipid metabolic process | 14 | 5.00E-06 |
| Lipid biosynthetic process | 13 | 4.40E-04 |
| Glycerophospholipid biosynthetic process | 6 | 4.60E-03 |

**337 genes - down with starvation only (anabolic bias)**

| **GO Term** | **Number of Genes/Count** | **P-Value** |
| --- | --- | --- |
| Metabolic process | 337 | 2.70E-81 |
| Macromolecule metabolic process | 211 | 5.50E-18 |
| Macromolecule modification | 66 | 1.30E-02 |
| Protein metabolic process | 115 | 1.50E-07 |
| Carbohydrate derivative metabolic process | 41 | 2.00E-07 |
| Nucleic acid metabolic process | 100 | 2.10E-07 |
| RNA metabolic process | 82 | 1.00E-06 |
| Regulation of RNA metabolic process | 34 | 1.60E-04 |
| RNA biosynthetic process | 39 | 1.60E+00 |
| mRNA metabolic process | 25 | 7.10E-05 |
| Cellular nitrogen biosynthesis process | 115 | 5.40E-23 |
| Regulation of nucleobase metabolic process | 36 | 9.20E-04 |
| Nucleoside phosphate metabolic process | 29 | 2.10E-07 |
| Organonitrogen compound catabolic process | 16 | 2.20E-03 |
| Histone modification | 8 | 3.10E-02 |
| small molecule metabolic process | 80 | 7.30E-15 |
| Co-factor biosynthetic process | 24 | 1.70E-06 |
