# Supplementary material for: mTORC1/AMPK responses define a core gene set for developmental cell fate switching
Source: BMC Biol. 2019 Jul 18;17:58. doi: 10.1186/s12915-019-0673-1 (PMC6637605; doi:10.1186/s12915-019-0673-1)
Supplement: Supplementary file 9 — Table S5. Signaling group. (DOCX 15 kb) [file 12915_2019_673_MOESM9_ESM.docx]

**Table S5**

**Signaling Group**

**138 genes - up with rapamycin/starvation**

| **GO Term** | **Number of Genes/Count** | **P-Value** |
| --- | --- | --- |
| Phosphorylation | 35 | 9.50E-16 |
| Protein phosphorylation | 31 | 4.50E-15 |
| Protein autophosphorylation | 3 | 4.60E-02 |
| Peptidyl-threonine phosphorylation | 2 | 9.80E-02 |
| cAMP biosynthetic process | 2 | 6.00E-02 |
| Chemotaxis | 6 | 5.00E-02 |
| Chemotaxis to cAMP | 5 | 5.00E-02 |
| Regulation of gene expression | 19 | 3.60E-11 |
| small GTPase mediated signal transduction | 18 | 4.10E-07 |
| Signal transduction | 16 | 1.90E-05 |
| Intracelluar signal transduction | 8 | 2.00E-04 |
| cAMP mediated signaling | 4 | 1.40E-02 |
| Adenylate cyclase-activating GPCR signaling pathway | 3 | 4.00E-03 |
| Phosphorelay | 4 | 1.10E-02 |
| Autophagy | 4 | 8.50E-03 |
| Developmental process | 14 | 1.70E-07 |
| Multicellular development | 10 | 2.40E-05 |
| Cell differentiation | 6 | 2.10E-03 |
| Aggregation | 9 | 9.20E-03 |
| Activation of MAPK activity | 5 | 8.20E-03 |
