# Supplementary material for: mTORC1/AMPK responses define a core gene set for developmental cell fate switching
Source: BMC Biol. 2019 Jul 18;17:58. doi: 10.1186/s12915-019-0673-1 (PMC6637605; doi:10.1186/s12915-019-0673-1)
Supplement: Supplementary file 10 — Table S6. Growth group. (DOCX 16 kb) [file 12915_2019_673_MOESM10_ESM.docx]

**Table S6**

**Growth Group**

**314 genes - down with rapamycin/starvation**

| **GO Term** | **Number of Genes/Count** | **P-Value** |
| --- | --- | --- |
| Ribosome biogenesis | 115 | 7.40E-110 |
| Ribosomal small subunit biogenesis | 31 | 3.50E-28 |
| Ribosomal Large subunit biogenesis | 29 | 3.80E-24 |
| Ribosome assembly | 14 | 1.80E-09 |
| Maturation of SSU-rRNA | 26 | 4.00E-26 |
| Maturation of 5.8S rRNA | 15 | 2.90E-14 |
| RNA processing | 100 | 9.70E-53 |
| rRNA processing | 91 | 6.10E-95 |
| ncRNA processing | 98 | 2.90E-77 |
| RNA modification | 26 | 9.00E-12 |
| tRNA modification | 7 | 4.10E-02 |
| tRNA processing | 12 | 1.20E-03 |
| Methylation | 34 | 6.20E-18 |
| RNA methylation | 15 | 8.30E-10 |
| Histone methylation | 4 | 1.10E-02 |
| Cell cycle | 42 | 9.00E-14 |
| Mitotic nuclear division | 29 | 4.10E-20 |
| Nuclear division | 31 | 1.40E-19 |
| Cell division | 23 | 1.30E+07 |
| Chromosome segregation | 14 | 7.90E-07 |
| Mitotic cell cycle | 34 | 2.40E-12 |
| Chromosome organization | 30 | 2.40E-10 |
| Organelle fission | 32 | 2.90E-18 |
| Chromatin organization | 14 | 8.80E-04 |
| Gene expression | 165 | 1.20E-61 |
| DNA replication | 31 | 1.20E-19 |
| Translation | 50 | 7.00E-18 |
| Transcription, DNA-templated | 23 | 8.10E-03 |
| DNA repair | 15 | 1.10E-02 |
