# Supplementary material for: mTORC1/AMPK responses define a core gene set for developmental cell fate switching
Source: BMC Biol. 2019 Jul 18;17:58. doi: 10.1186/s12915-019-0673-1 (PMC6637605; doi:10.1186/s12915-019-0673-1)
Supplement: Supplementary file 11 — Figure S5. Gene Ontology network analysis of genes regulated by starvation but not by rapamycin. A. Approximately 110 genes are induced and ~ 25 genes suppressed by starvation and not rapamycin with GO terms for developmental signaling. These were grouped for network association [45], with only minimal interactions seen. B. Approximately 120 genes are induced and 100 genes suppressed by starvation and not rapamycin with GO terms for growth. These were grouped for network association [45]. (PDF 762 kb) [file 12915_2019_673_MOESM11_ESM.pdf]

A.

SIGNALING

Up-Regulated by Starvation; No Rapa Effect

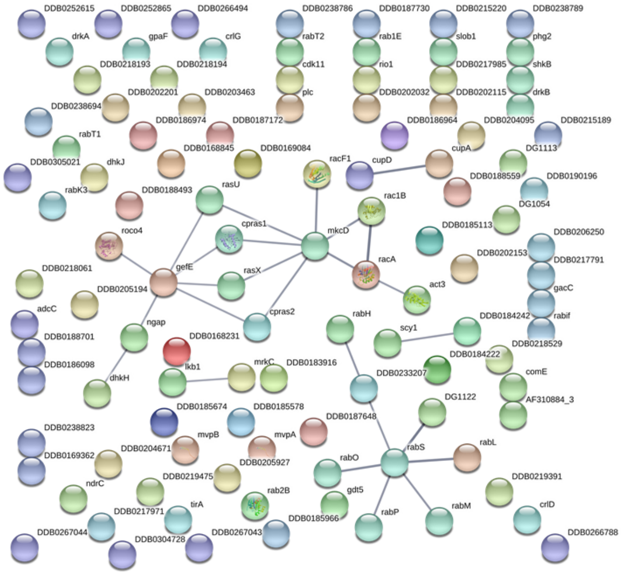

Down-Regulated by Starvation; No Rapa Effect

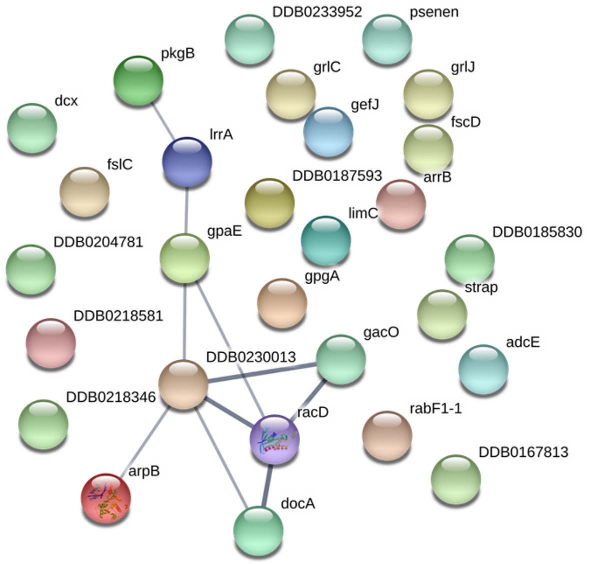

B.

GROWTH

Up-Regulated by Starvation; No Rapa Effect

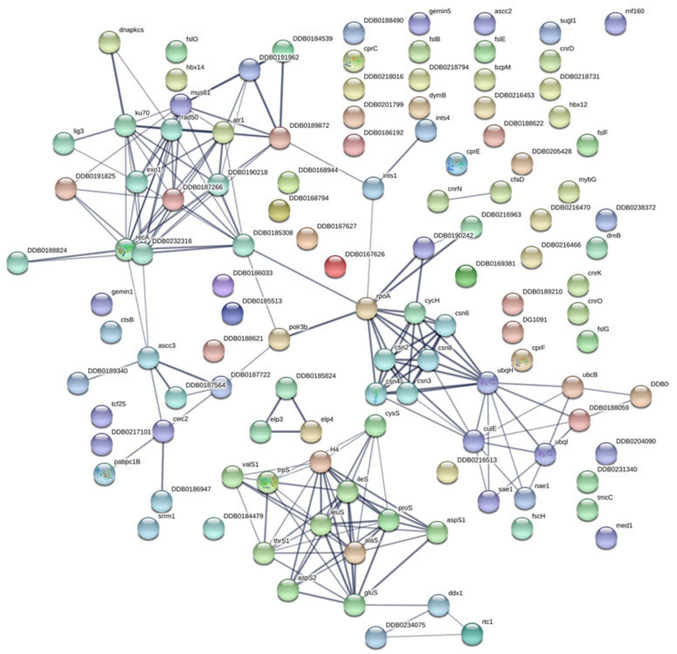

Down-Regulated by Starvation; No Rapa Effect

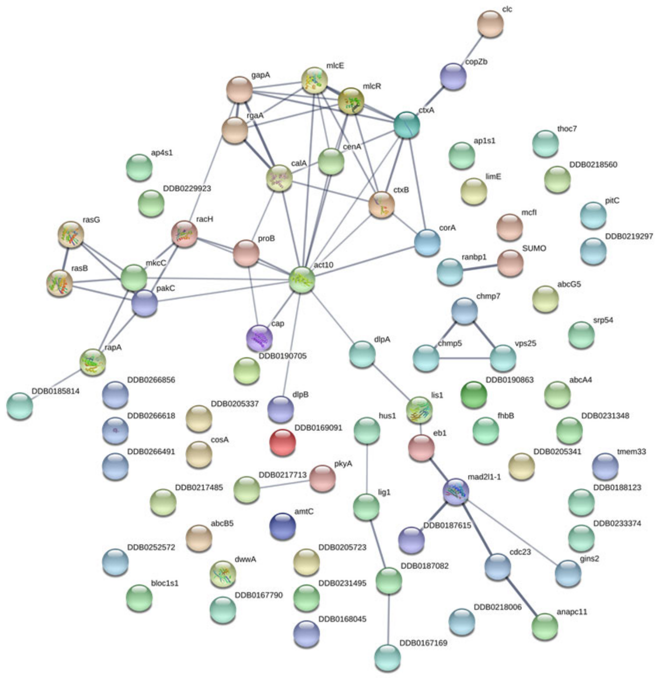

Supplementary Figure S5
