# Supplementary material for: mTORC1/AMPK responses define a core gene set for developmental cell fate switching
Source: BMC Biol. 2019 Jul 18;17:58. doi: 10.1186/s12915-019-0673-1 (PMC6637605; doi:10.1186/s12915-019-0673-1)
Supplement: Supplementary file 12 — Table S7. Secretion/uptake/transport group. (DOCX 17 kb) [file 12915_2019_673_MOESM12_ESM.docx]

**Table S7**

**Secretion/Uptake/Transport Group**

**47 genes - up with rapamycin/starvation**

| **GO Term** | **Number of Genes/Count** | **P-Value** |
| --- | --- | --- |
| Transport | 44 | 2.10E-14 |
| Vesicle mediated (endocyt./macropino.) | 29 | 1.20E-04 |
| Transmembrane | 10 | 5.40E-05 |
| Organic substance | 12 | 2.90E-04 |
| Protein | 8 | 1.00E-02 |
| Ion | 5 | 2.40E-02 |
| Localization | 47 | 1.70E-18 |
| Membrane organization | 4 | 5.70E-02 |

**85 genes - down with rapamycin/starvation**

| **GO Term** | **Number of Genes/Count** | **P-Value** |
| --- | --- | --- |
| Transport | 52 | 7.70E-06 |
| Vesicle Mediated (endocyt./macropino.) | 36 | 2.80E-02 |
| Transmembrane | 13 | 4.50E-05 |
| Organic substance | 15 | 1.20E-03 |
| Protein | 14 | 2.30E-04 |
| Ion | 4 | 5.30E-02 |
| Nucleocytoplasmic | 6 | 3.30E-03 |
| Localization | 62 | 1.60E-15 |

**214 genes - up with starvation only**

| **GO Term** | **Number of Genes/Count** | **P-Value** |
| --- | --- | --- |
| Transport | 148 | 2.20E-23 |
| Vesicle Mediated (endocyt./macropino.) | 106 | 6.30E-09 |
| Transmembrane | 32 | 1.50E-11 |
| Organic substance | 33 | 8.60E-06 |
| Protein | 19 | 1.80E-02 |
| Ion | 15 | 2.70E-04 |
| Lipid | 7 | 8.50E-04 |
| Secretion | 13 | 1.30E-05 |
| Localization | 148 | 1.00E-23 |
| Membrane organization | 10 | 6.10E-03 |

**279 genes - down with starvation only**

| **GO Term** | **Number of Genes/Count** | **P-Value** |
| --- | --- | --- |
| Transport | 196 | 5.50E-62 |
| Vesicle Mediated (endocyt./macropino.) | 196 | 3.70E-77 |
| Organic substance | 27 | 3.60E-03 |
| Protein | 23 | 1.60E-03 |
| Nucleocytoplasmic | 5 | 1.90E-02 |
| Secretion | 11 | 5.00E-04 |
| Localization | 196 | 3.30E-59 |
| Membrane organization | 13 | 6.00E-04 |
